# Monitoring the delicate operations of surgical robots via ultra-sensitive ionic electronic skin

**DOI:** 10.1093/nsr/nwac227

**Published:** 2022-10-21

**Authors:** Danyang Wei, Jiajie Guo, Yuqi Qiu, Shaoyu Liu, Jiangyan Mao, Yutian Liu, Zhenbing Chen, Hao Wu, Zhouping Yin

**Affiliations:** State Key Laboratory of Digital Manufacturing Equipment and Technology, School of Mechanical Science and Engineering, Huazhong University of Science and Technology, Wuhan 430074, China; State Key Laboratory of Digital Manufacturing Equipment and Technology, School of Mechanical Science and Engineering, Huazhong University of Science and Technology, Wuhan 430074, China; State Key Laboratory of Digital Manufacturing Equipment and Technology, School of Mechanical Science and Engineering, Huazhong University of Science and Technology, Wuhan 430074, China; State Key Laboratory of Digital Manufacturing Equipment and Technology, School of Mechanical Science and Engineering, Huazhong University of Science and Technology, Wuhan 430074, China; State Key Laboratory of Digital Manufacturing Equipment and Technology, School of Mechanical Science and Engineering, Huazhong University of Science and Technology, Wuhan 430074, China; Department of Hand Surgery, Union Hospital, Tongji Medical College, Huazhong University of Science and Technology, Wuhan 430022, China; Department of Hand Surgery, Union Hospital, Tongji Medical College, Huazhong University of Science and Technology, Wuhan 430022, China; State Key Laboratory of Digital Manufacturing Equipment and Technology, School of Mechanical Science and Engineering, Huazhong University of Science and Technology, Wuhan 430074, China; State Key Laboratory of Digital Manufacturing Equipment and Technology, School of Mechanical Science and Engineering, Huazhong University of Science and Technology, Wuhan 430074, China

**Keywords:** surgical robots, delicate operations, ultra-sensitive, ionic electronic skin, tactile sensing

## Abstract

The arrival of surgical robots in high-end medical equipment is a landmark, and the realization of tactile sensation a major challenge in this important cutting-edge research field. Aiming to address this issue, we present ultra-sensitive ionic electronic skin in the form of flexible capacitive pressure sensors, which incorporate multistage bionic microstructures in ion gels for the purpose of monitoring the delicate operations of surgical robots. Significantly, the ionic skin exhibits an ultra-high sensitivity of 9484.3 kPa^−1^ (<15 kPa), and the sensitivity remains higher than 235 kPa^−1^ in the wide range of 15–155 kPa. The device has also achieved a detection limit as low as 0.12 Pa or, equivalently, 0.31 mg, fast response within 24 ms, and high robustness (loading/unloading for 5000 cycles without fatigue). The sensor facilitates the challenging task of tele-operated robotic threading, which exceeds the human tactile perception limit when threading a needle. We have also confirmed that ionic skin can be used in robot-assisted invasive surgery, such as incision/resection of tissues and suturing of wounds, providing tactile information to surgeons to improve operation success rates. The flexible ionic skin is capable of conforming to the various shapes of robotic manipulators, thus has great promise for applications in robotic dexterous manipulation, prosthetics and human–machine interfaces.

## Teaser

An ultra-sensitive ionic electronic skin is utilized for robot-assisted invasive surgery, such as incision/resection of tissues and suture of wound, to provide tactile information to surgeons to improve the operation success rates.

## INTRODUCTION

Medical equipment is critical for healthcare and health promotion [1,2]. As a landmark of high-end medical equipment and important cutting-edge research fields today, surgical robots are having a revolutionary impact on disease diagnosis [3], surgery [4–8], rehabilitation [7,8] and medical services [9], and are leading the development of precision medicine, telemedicine and smart medicine [3–9]. Compared with traditional manual surgery, robot-assisted surgery has higher operation precision and better operation quality; this has been demonstrated in neurosurgery [4,10], cardiothoracic surgery [11], gynecology [12,13], urology [5,8] and other fields [1,3,6,7,14]. It has been pointed out in surgical research reports that the robot-assisted method can improve medical surgeries through a remote control system [1,3,4], thus increasing surgical efficiency, reducing operation complications and relieving surgeons’ fatigue [12]. However, the existing surgical robots, such as Da Vinci robot, only have image feedback without any intraoperative sense of touch [14–16]. Because of this, surgeons can only manipulate the end-effectors by remotely operating the joystick and watching the images transmitted from the end-effector during surgery, but cannot identify the tissue attributes and tissue lesions through relevant tactile data. This increases uncertainty and risk in the surgery [10,15,16]. Haptic sensing is the key to tackling these challenges [17], and the implementation of tactile sensors on surgical robots is desired for force feedback control [14–16].

To address these issues, some advanced pressure-sensing mechanisms, such as piezoresistive [18,19], capacitive [20,21], magnetic [22] and optical [23] mechanisms [24,25], have been explored for tactile sensors. For instance, Hidaka *et al*. simulated the tactile sensation process of human hands and made a ‘finger-like sensor’, which could measure various types of tactile information such as roughness, hardness and the texture of an object’s surface [26]. Kim *et al*. put forward a new surgical instrument with a four-degrees-of-freedom force sensor, which combined four capacitive sensors with a customized surgical instrument to sense the normal force and shear force at the tip [27]. However, most pressure sensors developed were not flexible or stretchable, thus it was difficult to integrate them onto robotic manipulators with a high degree of freedom. With the recent development of flexible and stretchable electronics, electronic devices mimicking the comprehensive tactile sensing capability and mechanical properties of human skin, i.e. Electronic Skin (e-skin) [28], can detect and monitor proximity, pressure, strain, temperature, etc. Shen *et al*. prepared a pressure-sensitive robotic skin that can provide an industrial robot and a transradial amputee with real-time sensory feedback, facilitating dexterous manipulation and secure interactions with the external environment [20]. Chang *et al*. proposed a collision-aware flexible piezoresistive sensor for real-time monitoring of the contact between a surgical robot and human tissue [19]. However, there are still some limitations in those flexible sensors, such as a small pressure range, low sensitivity, complicated manufacturing processes, and slow response or poor stability, which have to be overcome to realize fine and intelligent operations carried out by a surgical robot.

The bio-inspired design has become an attractive strategy to improve the sensitivity of flexible tactile sensors [21,29]. Herein, we report a new type of capacitive ionic e-skin, which was inspired by the multilevel microstructure on the pollen surface of *Myosoton aquaticum* and can be used as ultra-high-sensitivity ionic e-skin for surgical robot tactile sensing. This multilayer microstructure consists of a micro-pit structure and micro-cone structure, enabling ultra-high sensitivity in a large pressure measurement range of the ionic skin. Specifically, we have studied the sensory organs of *M. aquaticum*, investigated the physical characteristics of functional materials, reproduced the microstructure of the pollen surface, and established the corresponding structural model. Through the optimization of process parameters based on characteristics of ionic gels, and by replicating natural micro-cone structures on the surface of *Calathea zebrine* leaves, the ultra-high-sensitivity flexible capacitive ionic skin simulating multistage bionic microstructures (MBMs) on the surface of *M. aquaticum* pollen can be prepared. The electronically conductive composite (ECC) adopted in this sensor as electrode materials have good conductivity and stretchability, and can be fabricated into a large-area array at low cost, and maintain excellent performance on complex curved surfaces. The unique characteristics of the electric double layer (EDL) at the capacitor interface endow the ionic skin with ultra-high capacitance per unit area and thus extra-high pressure sensitivity that is >1000 times larger than that of traditional solid-state capacitive sensors. Because the micro-cone and micro-pit structures increase the contact area between the electrode and the dielectric layer surface, the ionic skin exhibits a highest sensitivity of 9484.3 kPa^−1^ in the low-pressure regime (0–15 kPa), and the sensitivity is still above 235 kPa^−1^ in the broad range of 15–155 kPa. The sensor has achieved a limit of detection (LOD) as low as 0.12 Pa and a fast response time (within 24 ms), and can detect ultra-light objects with a weight of 0.31 mg. It also shows high stability: over 5000 loading/unloading cycles without fatigue. The proposed flexible ionic skin can be easily attached to various robot manipulators. Due to its ultra-high sensitivity, the sensor facilitates the challenging task of tele-operated robotic threading, exceeding the human tactile perception limit when threading a needle. Monitoring the proximity of adjacent objects, and perceiving the incision/resection of tissues and suturing of wounds, which are critical to robot-assisted invasive surgery, are also demonstrated by the ionic skin. With the high sensitivity enabled by bio-inspired structure design, MBM-based ionic e-skin sensors are promising for applications in robotic dexterous manipulation, prosthetics and human–machine interfaces.

## RESULTS AND DISCUSSION

### Design and fabrication of ionic skin with an MBM dielectric layer

**Figure 1.**
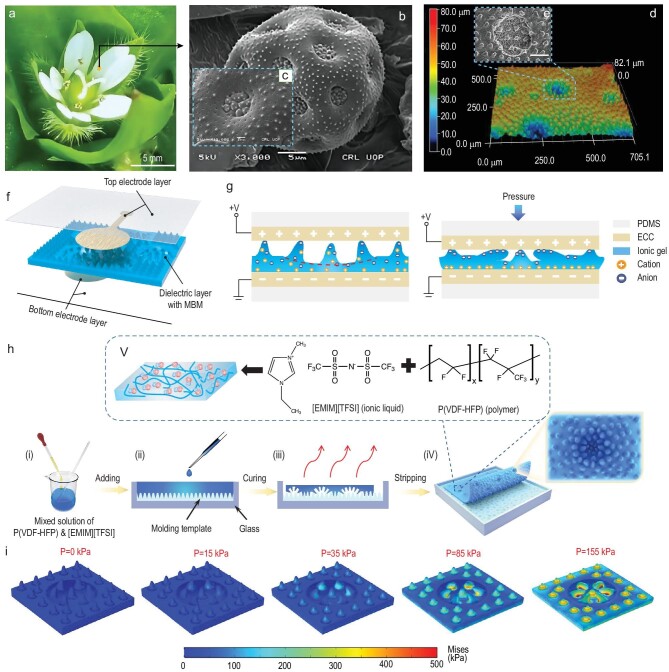
Sensitive design of capacitance sensor dielectric layer surface microstructures inspired by *Myosoton aquaticum* pollen surface multistage microstructures. (a) Photograph of *M. aquaticum* (Caryophyllaceae). (b and c) Scanning electron microscopy (SEM) micrographs of the pollen of *M. aquaticum* reproduced from Ullah *et al* [30]. (b) *M. aquaticum* pollen grain (bar = 5 μm) [30]. Copyright 2018, Wiley-VCH. (c) SEM showing detail of exine sculpture at aperture (bar = 1 μm) [30]. Copyright 2018, Wiley-VCH. (d) 3D surface topography of the prepared dielectric layer. The surface of that dielectric layer film is featured with micro-pit-shaped structures and micro-cone-shaped structures at different scale levels, wherein the micro-pit-shaped structures are randomly distributed, and the micro-cone-shaped structures are uniformly distributed on the surface of the film, including the micro-pit-shaped structures. (e) SEM image of the prepared dielectric layer showing details of the micro-pit structure demonstrates that micro-cones are also distributed in the micro-pit-like structure (bar = 50 μm). (f) Schematic diagram of the MBM-based ionic electronic skin sensor structure. (g) Schematic illustration for the functioning of the ionic e-skin sensor before and after applying pressure. Graphical illustration of the sandwich structure and charge distribution in the sensor. The red dotted line depicts the micro-pit structure. (h) Schematic illustration of the fabrication processes of an MBM dielectric layer. Inset (V) depicts the molecular structures of the matrix and the IL : [EMIM] [TFSI] and P(VDFHFP). (i) Stress distribution of the finite element model under different external loads.

The surface of pollen has a micro-nano gradient structure with complex morphology, which varies with different species, and it is an excellent template for constructing a micro-nano gradient structure with special functions [29]. For the capacitive pressure sensor, the presence of a microstructure in the dielectric layer can reduce the viscoelasticity of the polymer material, thus shortening the recovery time of deformation and improving the response speed of the sensor [21]. The existence of the air gap between microstructures reduces the overall elastic modulus of the dielectric layer, rendering it more deformable by external pressure, thereby improving the sensitivity of the sensor [25]. At present, a new strategy is needed in order to create a capacitive sensor with high sensitivity over a wide pressure range. As shown in Fig. 1, *M. aquaticum* belongs to Caryophyllaceae and its pollen grain surface has a unique multistage and multiscale microstructure with a plurality of micro-pit-like structures with pit diameters of ∼6.0–7.0 μm, and micro-cone structures evenly distributed in the whole pollen sphere, including micro-pits [30,31]. Inspired by this unique microstructure, we have studied and prepared the MBM dielectric layer, whose three-dimensional surface morphology is shown in Fig. 1d and e. The details of the micro-pit structures on the MBM dielectric layer are randomly distributed, and the micro-cone structures uniformly cover the surface of the whole film. The cross-sectional dimensions of the dielectric layer are displayed in Fig. S2. The pit-like structure on the surface of the dielectric layer has a depth of ∼30 μm and a diameter of ∼45–300 μm, and the average height of the micro-cones on the surface of the dielectric layer is ∼24.6 μm. The average bottom diameter is ∼20.4 μm, and the average inter-cone distance is ∼33.6 μm. The multilevel microstructure on the surface of the dielectric is similar to that on the pollen surface of *M. aquaticum*, and will substantially improve the pressure sensitivity of the capacitive e-skin sensor.

The structure of the e-skin sensor is depicted in Fig. 1f. The sensor is composed of multilayer scalable structures and functional materials, rendering the overall device highly scalable. Based on our previous work [32], stretchable ECCs are adopted as the top and bottom electrode materials and polydimethylsiloxane (PDMS) is chosen as the flexible substrate of the ECC. The manufacturing process for the electrode layers is shown in Fig. S3 and the details are in the supplementary data. The ionic gels function as the dielectric layer and the extraordinary ionic-electric capacitive interfaces contribute to the remarkably high change in capacitance compared to parallel-plate capacitors, upon deformation. As illustrated in Fig. 1g, when the ionic gel film is in contact with the ECC electrode, the electrons on the ECC surface repel the negative ions and attract the positive ions in the ionic gel at the same time, and the ionic gel film and the released ECC surfaces form a unique ion-electron contact on each side to produce the interface capacitance of the EDL. The unique characteristics of the EDL at the capacitor interface endow the ionic skin with ultra-high unit-area capacitance and thus force–capacitance change, which is >1000 times higher than that of traditional solid-state capacitive sensors [33]. With a constant sensor thickness, the capacitance is proportional to the EDL area. The initial capacitance of the sensor is quite low in the absence of external loads, because the formed EDL area is small due to the small number of micro-cones in contact with the electrodes and the small contact area of the ion-electron interfaces. Under a low external pressure, the capacitance change rate significantly increases as the MBM on the ionic gel film experiences mechanical deformation, with a dramatic increase of the EDL area. According to the Gouy-Chapman-Stern model [34], this interface without electrochemical activation can be simplified as a capacitive element. The equivalent circuit of the whole device is described by Fig. S4 and detailed discussion is shown in the supplementary data.

In order to mimic the multilevel microstructure of the pollen grain surface of *M. aquaticum*, a low-cost fabrication method is adopted as shown in Fig. 1h. The ionic gel uses a blended solution of poly(vinylidene fluoride-co-hexafluoropropylene) (P(VDF-HFP)) as the structuring polymer and 1-ethyl-3-methylimidazolium bis(trifluoromethylsulfonyl)imide       ( [ EMIM ] [TFSI]) as the ionic liquid (IL). The micro-cone structure of the pollen surface is similar to that of *C. zebrine* leaves, so the micro-cone structure of the *C. zebrine* leaf surface is replicated by PDMS as the preparation template, as shown in Fig. S5, and then the ionic gel mixed solution is dripped onto the PDMS surface, and the whole surface of the PDMS template is heated at a temperature of 26°C–40°C and humidity of 60%RH–99%RH. During the preparation of ion gel solution, a large amount of air bubbles will be generated due to continuous stirring by the magnetons, especially micro bubbles. At high ambient humidity, the bubbles will expand due to a higher temperature during curing. Meanwhile, P(VDF-HFP) dissolves in acetone solution, and the ionic gel mixed solution will shrink during curing due to its volatile characteristics, as shown in Fig. S6. During curing at higher temperature, the existence of micro-cone structures will render the thermal stress between different materials uneven, and the surface binding energy in different regions is different. Therefore, these bubbles will vaporize along with the ion gel, and form randomly distributed bubble points on the surface of the film, thus the randomly arranged micro-pit structure appears. A finite element model (FEM) is built to investigate the sensing mechanism of the MBM-based flexible capacitance sensor, as shown in Fig. 1i (see the supplementary data for details). The simulation results illustrate four stages of microstructure deformation. In the low-pressure regime, the existence of an MBM increases the rate of change of contact areas, playing a significant role in improving the pressure measurement sensitivity. Under high pressure, the deformation of the MBM structure approaches the limit, and the variation of pressure sensitivity depends on the thickness variation of the dielectric layer. Detailed analysis of deformation and capacitance change in the four stages can be found in the supplementary data.

### Characterizations of sensing materials and microstructures

**Figure 2.**
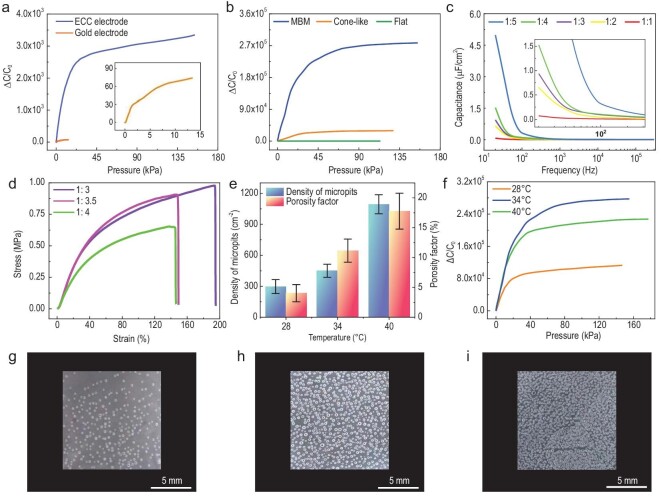
Characterizations of sensing materials and influence of microstructures on sensor performance. (a) Comparisons of the pressure measurement performance of sensors with the ECC electrode and these with the gold thin-film electrode at a test frequency of 300 kHz. In the inset, the enlarged view of lower pressure is displayed. (b) Comparisons of the performance of sensors with different dielectric layer surface microstructures. (c) The frequency dependence of area capacitance with different PVDF-HFP : [EMIM] [TFSI] ratios. The enlarged view of the plot at lower frequency is shown in the inset. (d) The tensile stress-strain curves of PVDF-HFP : [EMIM] [TFSI] with ratios of 1 : 3, 1 : 3.5 and 1 : 4. (e) Density and porosity of the micro-pit structures of MBM films prepared at different temperatures. The average density represents the average number of micro-pit structures per square centimeter. The porosity represents the percentage of the whole film area that micro-pit structures account for. (f) Capacitance changes as a function of applied pressure under different IL concentrations. Photographs of MBM films prepared at (g) 28°C, (h) 34°C and (i) 40°C.

This section investigates influential factors on e-skin sensor capacitance, including ion concentration, conductive materials of the electrode layer and microstructures prepared under different process parameters. Some observations can be made from the results shown in Fig. 2:

- Gold thin-film electrodes are common flexible electrodes used in flexible sensors. To compare the sensing performance of ionic skin prepared by ECC electrodes and gold thin-film electrodes, Fig. 2a shows that the pressure range and sensitivity of ECC ionic skin are more than 10$\times $ and 431$\times $ higher than those of ionic skin using gold electrodes.
- We performed finite element simulation to compare the measurement performances of sensors with micro-cone microstructures and multistage microstructures. As shown in Fig. S7, under the same applied pressure, the displacement between the top and bottom electrode of the sensor with an MBM structure is larger, thus leading to larger capacitance change than the sensor with only a cone-like microstructure. Therefore, the MBM structure can increase the measurement sensitivity of the e-skin sensor. Figure 2b shows the comparisons of three dielectric layer designs, including the flat surface, micro-cone structure and the MBM dielectric layer, and the pressure sensitivity and sensing range of the MBM design is much higher than those of the other two.
- Figure 2c shows that the unit-area capacitance of ionic gel is highly frequency dependent, and the decreasing rate is high at low frequency. In brief, a higher IL ratio leads to a larger interface capacitance, which leads to a higher measurement sensitivity.
- Furthermore, we compare the tensile properties of flat dielectric films with PVDF-HFP : [EMIM] [TFSI] ratios of 1 : 4, 1 : 3.5 and 1 : 3. As shown in Fig. 2d, the ultimate strain of the films decreases with the increase in weight percentage of ionic liquids and the tensile properties of 1 : 4 and 1 : 3.5 are close. Compared with the films with a ratio of 1 : 3, the unit-area capacitance-frequency curves of the films with a ratio of 1 : 4 and 1 : 3 are similar, and the capacitance per unit area of the films with a ratio of 1 : 4 only increases by ∼2% at the frequency of 1 kHz, as shown in Fig. 2c. The capacitance per unit area with a ratio of 1 : 3.5 is almost as high as that of 1 : 4. Notably, a different curing temperature would produce different micro-structures on the surface of the prepared MBM dielectric layer, as shown in Fig. 2e. The capacitance change of ionic skin is shown in Fig. 2e. The samples of MBM films were prepared at 28°C, 34°C and 40°C, and the corresponding average densities of micro-pit structures were counted in scanning electron microscopy (SEM) images as 297/cm^2^, 450/cm^2^ and 1094/cm^2^, respectively. The porosity of the micro-pit structures of MBM films was 4.04%, 11.16% and 17.77% for 28°C, 34°C and 40°C, respectively, which indicates that a higher ambient temperature produces larger effective sensing areas of the micro-pit structures.
- The change range of the capacitance pressure of ionic skin can be divided into four stages, which is consistent with previous simulation (Fig. 2f and Fig. 1i). In the low-pressure area, the sensitivity of ionic skin is extremely high, as shown in Fig. 2f. The sensitivity of ionic skin prepared at 28°C is lower, at 6117.8 kPa^−1^ (Fig. S8). The sensitivity of ionic skin prepared at 34°C and 40°C is similar, and the sensitivity of ionic skin prepared at 40°C is slightly higher. From the second stage to the fourth stage, the sensitivity of the ionic skin prepared at 34°C is obviously higher than that of the ionic skin prepared at 40°C.
- In order to further study the influence of temperature on the micro-pit structure, we counted the diameters of micro-pit structures on the films prepared at each temperature (Fig. 2g–i) through observation by SEM; they are close to normal distribution (Fig. S8). When the temperature is 28°C, the average diameters of micro-pits are 123.73 μm, compared to the average diameters of 156.77 μm and 117.63 μm obtained at 34°C and 40°C, respectively.

As demonstrated in Fig. 2a, due to the insufficient adhesion between the gold thin-film and PDMS substrate, the bonding is easy to break. When the applied pressure exceeds 13.37 kPa, the deposited gold falls off from the dielectric film, and the surface of the gold electrode cracks easily, leading to the failure of the sensor. In previous work, the electrical conductivity of the ECC material in the unstretched state was as high as 1.21 × 10^4^ S/cm, and the stretchability could reach 119%. When the ECC material is stretched to 30% strain, its electrical conductivity is in the same order of magnitude as that in the unstretched state, and its initial electrical conductivity does not change after 100 reciprocating cyclic stretches. It can be seen that the high conductivity and stretchability mean ECCs are excellent materials for electrodes of MBM e-skin sensors.

It is noted that the high proportion of ionic liquid leads to a low elastic modulus and structural stability of the film. With the increase in proportion of ionic liquids, the films become softer, and the hysteresis of the devices becomes more obvious, which renders it difficult to maintain the structural integrity of the devices under high pressure, and it is also difficult to ensure the mechanical flexibility of ionic gel films. The images of ionic gel films prepared in different proportions (Fig. S9) show that the composite ratio of 1 : 5 is not suitable for device fabrication due to the loss of mechanical integrity. And the improved sensitivity is due to the deformation mechanism of the contact area contributed by the low density of micro-pits, large pit diameter and numerous micro-cones in the micro-pit structure of the MBM film. In subsequent performance tests, the ionic gel solution was prepared with a P(VDF-HFP) : [EMIM] [TFSI] ratio of 1 : 3.5, and the MBM film as the dielectric layer of ionic skin was prepared by controlling the ambient temperature at 34°C, which not only ensured a large capacity per unit area of the ionic gel membrane, but also maintained a certain amount of mechanical flexibility.

### Measurement performance of the ionic e-skin sensor

**Figure 3.**
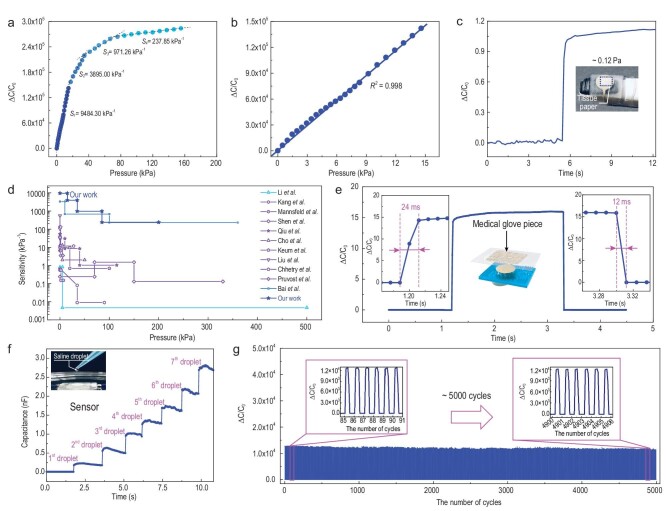
Measurement performances of MBM-based ionic e-skin sensors. (a) Change in capacitance as a function of pressure in the range of 0–155 kPa. (b) The capacitance change in the first stage (0–15 kPa) has high linearity. (c) Detection of 0.31 mg of 5 mm × 5 mm single-layer light tissue paper using an 8 mm diameter MBM sensor unit. The corresponding average effective pressure is ∼0.12 Pa. (d) Comparisons of the sensitivity of our pressure sensor with previously reported tactile sensors for robots. (e) Pressure response of the sensor to a medical glove piece placed on top. Insets show response and recovery time. (f) Measurements of saline drops. (g) Stability of the sensor tested for 5000 compression/release cycles under a pressure of 1 kPa.

The sensing characteristics of MBM ionic skin are evaluated in detail. As shown in Fig. 3a, it can be observed that the ionic e-skin sensor based on an MBM dielectric layer has a high sensitivity of 9484.30 kPa^−1^ in the first stage (0–15 kPa, Fig. 3b), 3895.00 kPa^−1^ in the second stage (15–35 kPa), 971.26 kPa^−1^ in the third stage (35–85 kPa) and 237.85 kPa^−1^ in the fourth stage (85–155 kPa). Pressure sensitivity can be defined as $S = \delta ( {\Delta{C} /{C}_0} )/\delta P$, where *P* is the applied pressure, and $\Delta C$ and ${C}_0$ refer to the change of capacitance and the initial capacitance without loading, respectively. Figure 3b shows the unprecedented high sensitivity and linearity of the sensor in the range of 0–15 kPa, justifying its application in tiny pressure detection, which is further illustrated in Fig. 3c where the drop of a 0.31 mg tissue paper is detected via an effective pressure change of 0.12 Pa (Fig. S10) with smooth response and no fluctuation in the presence of environmental interferences. In addition, the rapid response of the MBM sensor is also observed over a wide range of applied pressures (6, 20, 66, 155 kPa), as shown in Fig. S11. Compared with the capacitive e-skin results reported in previous studies [20,21,35–44], our ionic skin has the highest sensitivity at 9484.3 kPa^−1^ in the range of 0–15 kPa, as shown in Fig. 3d and Table S1 in the supplementary data. It is worth noting that our sensor still shows high sensitivity in the range of 15–155 kPa.

As shown in Fig. 3e, the ionic skin responds to the touch and sudden release of a medical glove piece within 24 ms and 12 ms, respectively, which is faster than the human skin response of 30–50 ms. In Fig. 3f, the ionic skin accurately detects sequential applications of seven saline droplets (20 μl for each droplet) with large capacitance response on the scale of nF. The flexibility and stretchability of the sensor are very important for its application. Static bending experiments and cyclic dynamic bending experiments using ionic skin with different bending radii were performed, respectively, as shown in Fig. S12. It can be seen from Fig. S12a that the capacitance output signal gradually increased as the bending radius decreased from 22 mm to 11 mm. At a constant bending radius, the signal is found to be quite stable after many bending cycles (Fig. S12b). We also studied the pressure response of the sensor under tension, as shown in Fig. S13. Although parallel-plate capacitors are sensitive to humidity and an EDL is susceptible to ambient temperature, the properly packaged sensor is almost undisturbed under a pressure of 10 kPa in an environment humidity of 24%RH, 62%RH and 99%RH, as shown in Fig. S14a. Besides, the response of the device is stable at a given temperature, although it varies at different temperatures, indicating that sensor calibration is necessary to eliminate temperature effects in extreme cases (Fig. S14b). It was noted that different batches of sensors prepared under the same preparation conditions also maintained excellent measurement performances (Fig. S15). Moreover, the sensor durability and robustness were evaluated by 5000-cycle tests of loading and unloading at 1 kPa (Fig. 3g), where no drift in response was observed and almost the same amplitude and waveform were maintained throughout the process, confirming the high durability and robustness of the sensor.

### Ultra-high sensitivity of the e-skin sensor for needle-threading tasks

At present, manual threading of suture needles, which has been a major obstacle to improving surgery efficiency, is a time-consuming and laborious task in clinical practice. The rapid threading of a surgical needle using a robotic manipulator is a great challenge, as the tiny pressures of needle–thread interactions are hardly detectable, and computer vision may not be effective in detecting actual contact of needle and thread. The proposed MBM sensor with ultra-high measurement sensitivity to pressure changes is a competent candidate for robotic needle-threading tasks.

**Figure 4.**
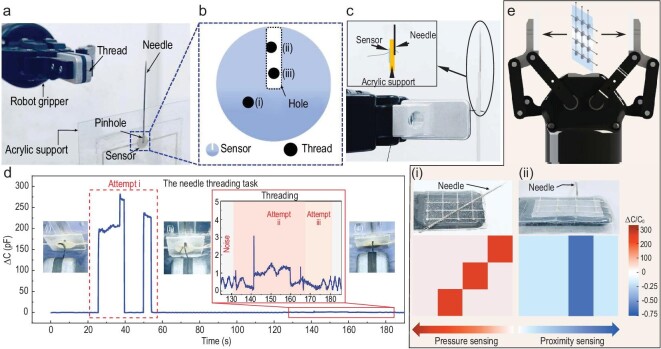
The tele-operated needle threading task. (a) Schematic illustration of experimental set-up. The suture needle is fixed in the acrylic support plate with the pinhole aligned with the sensor hole, and the robotic gripper holds the suture to complete the task. (b) Enlarged view of the structure of the sensor, and the possible relative position with the suture in the experiment. The blue part is the sensing area, and the hole is the area where the suture passes through. (c) Positions of thread, needle and acrylic support, and photo of successful needle threading. The diameter of this needle is 0.9 mm. (d) Real-time response of the MBM-based sensor during three threading attempts. When the suture is not inserted into the pinhole and touches the sensing area of the sensor, the sensor immediately shows obvious response. When threading starts, a low response from the sensor indicates the beginning of threading. The photo in the figure shows three positions of the suture and sensor. (e) The integration of the sensor array onto a robotic gripper. The upper pictures of (i) and (ii) show that the needle is placed on the sensor array and the needle is 1 cm away from the sensor array, respectively. The lower pictures show the pressure mapping of the needle weight (left) and the 3D shape mapping of the needle corresponding to the top picture (right).

Here, we demonstrate scenarios in which a robotic gripper threads a soft suture (diameter of 0.1 mm) through the eye of a needle, and the MBM sensor provides the tactile signal for feedback control during the process. Figure 4a shows the experiment set-up for the task, in which a slot was laser cut on the MBM sensor (Fig. 4b) to hold the surgical needle (with an eye diameter of 0.7 mm) in the acrylic support (Fig. 4c). There are three cases in the threading task, as illustrated in Fig. 4b: (i) the suture is outside the eye of the needle; (ii) the suture touches the edge of the eye but has not been fully inserted into it; (iii) the suture successfully passes through the hole without making contact with the edge. Figure 4d illustrates the robotic threading process, where initially the suture hits the sensing area outside the hole twice (Case (i)), producing a large response with the maximum $\Delta C$ > 250 pF. From 25 s to 40 s, the measured capacitance further rises because the suture continues to move forward after contacting the acrylic. When the manipulator retreats and the suture is separated from the acrylic, the pressure quickly drops to zero. As the adaptive gripper continues to adjust the suture position, the sensor captures the sinusoidal vibration of the machine table (Fig. 4d inset). Since the laser cutter cut the sensing part of the MBM sensor directly and exposed the electrode at the edge of the sensor hole, the suture contacts and rubs against the exposed electrode when it touches the needle edge, and the slight displacement of the suture advancement results in a spike in measured capacitance (Case (ii)). As this happens at the sensor edge incidentally, the impulse pattern is different from the continuous changing signal in Case (i). As the robot further adjusts the threading motion, the signal gradually returns to the initial state when the threading task is completed (Case (iii)). Supplementary Movie S1 presents the remote control of the robot and the monitoring signals of the MBM sensor during the needle-threading process. Different threading states can be identified by the signal patterns to facilitate the automatic suture operations of a surgical robot.

A sensor array consisting of 3 × 4 MBM e-skin sensors on the two-fingered manipulator can detect pressure distribution when handling needles and needle proximity (Fig. 4e). As shown in Fig. 4e(i), when a needle is placed on the sensor array, the spatial distribution of pressure indicates the needle position and orientation. The working principle of the sensor for proximity detection is explained in Fig. S17a. Figure 4e(ii) shows that the needle positioned 1 cm above the third column (from left to right) of the sensor array leads to a decrease of 0.39 pF in the measured capacitance (a change of −32.3%). Motion tracking of a surgical needle through the sensor array, when the robotic manipulator moves, is illustrated in Fig. S17b. Overall, capacitance mapping of the sensor array demonstrates increased capacitance for pressure distribution sensing and reduced capacitance for needle proximity estimation.

### Monitoring needle insertion for suturing operations

Suturing, as one of the most common surgical tasks, is essential to ensure good healing [45]. Therefore, robotic autonomous suture technology has been widely studied [45,46]. Suturing consists of three processes—inserting needles, pulling needles and pulling wires—which are challenging for an autonomous robot to achieve with precision [46]. The needle insertion position is particularly critical to the safety of surgery, as accidentally touching other tissues may cause massive bleeding, and the penetration depth has to be precisely controlled to avoid additional wounds and facilitate healing. Thanks to its ultra-high sensitivity, the e-skin sensor can be utilized to monitor the delicate operation of needle insertion.

**Figure 5.**
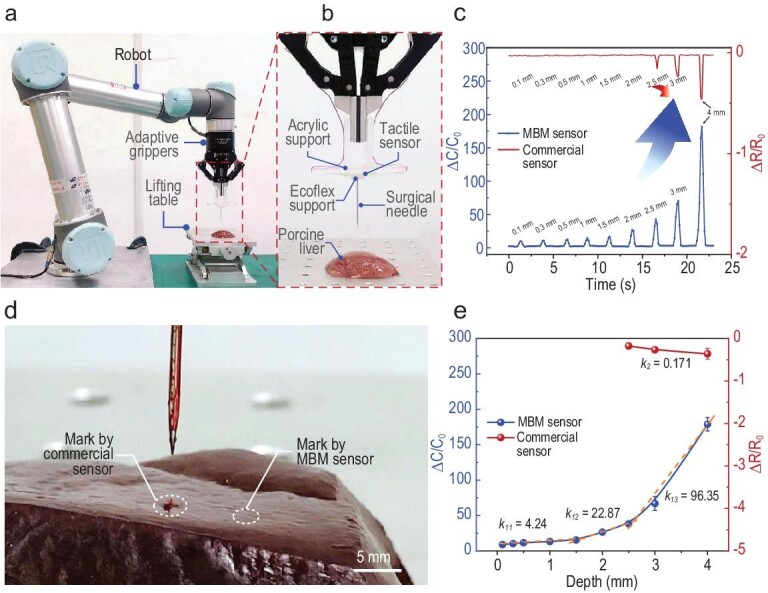
Monitoring of needle insertion operations by a robotic manipulator. (a) The experimental set-up. (b) Zoomed-in view of a sensor unit mounted on the manipulator for needle insertion monitoring. (c) Real-time monitoring of capacitance variations of the sensor installed at the bottom of the needle, and comparisons with commercial sensors. Penetration distances under 2.5 mm cannot be detected by the commercial sensor. (d) The penetration marks by the needle when the commercial sensor and MBM sensor just identify the contact between the needle and tissue. During the experiment, the needle moved towards the tissue until a noticeable change in $\Delta X/{X}_0$ (resistance/capacitance) occurred, followed by the retreat of the needle. (e) Sensitivity, with regard to measuring penetration depth, of the MBM sensor and the commercial sensor.

Figure 5a shows the experimental platform for the monitoring of robotic needle penetration into tissues. The sensor unit is installed on the two-fingered robotic manipulator via an acrylic support, and the surgical needle is supported by Ecoflex soft substrate so that the touching force is transmitted to the sensor without interference from other forces, as detailed in Fig. 5b. Because porcine liver is similar to human internal organs in terms of mechanical properties [47], and because it is softer and thus more challenging for suturing and resection than skin tissues, porcine liver tissues are adopted in this experiment to better demonstrate the high performance of the e-skin sensor. The contact force on the tissue sample by the needle is converted into normal pressure to the sensor unit. The depth of needle insertion is characterized by the height of the lifting platform, and an automatic control program is adopted to control the two-fingered robotic manipulator to move up and down at the same speed.

We compare the performance of the ionic e-skin sensor with a widely used commercial resistive pressure sensor, as shown in Fig. 5c. When the needle tip just touches the tissue sample, this extremely small distance can be detected by the MBM sensor while the commercial thin-film sensor has no notable response. When the lifting platform rises by as much as 2.5 mm, this displacement has already caused a large needle penetration mark on the surface of the tissue, and the commercial sensor only produces a barely notable resistance change. In contrast, our MBM sensor has already produced a response at ∼0.1 mm penetration, and at this distance the needle has not damaged the tissue, as shown in Fig. 5d. For the purpose of comparison, we define the sensitivity to measure penetration depth as $k = \delta ( {\Delta X/{X}_0} )/\delta d$, where *d* is the penetration depth, and $\Delta X$ and ${X}_0$ refer to the change of resistance/capacitance and the initial resistance/capacitance without loading for the commercial sensor/MBM sensor, respectively. The MBM sensor capacitance increases monotonically with the needle insertion depth, as shown in Fig. 5e, and the sensor sensitivity reaches 4.24 mm^−1^ in the insertion range of 0–1.5 mm, 22.87 mm^−1^ in the range of 1.5–2.5 mm, and 96.35 mm^−1^ above 2.5 mm, while that of commercial sensors is only 0.171 mm^−1^ in the range of 2.5–4 mm. The MBM sensor has higher sensitivity and a wider detection range than commercial sensors; these attributes are essential for monitoring needle advancement in ultra-delicate operations in target tissues and for avoiding accidental damage to other regions during surgery. We further investigate the performance of the MBM sensor and the commercial sensor during needle insertion into even softer tissues such as the brain, as shown in Fig. S18. Bean curd is adopted for the experiment due to its similar mechanical properties. It is worth noting that this sensor can also sense the extremely small needle penetration distance on soft bean curd, which verifies the high sensitivity of this sensor and its potential application in brain surgeries. Due to the ultra-high sensitivity, the MBM sensor can monitor not only needle insertion and exit in tissue, but also the low magnitude force caused by fine movement in the tissue, providing valuable tactile feedback for precision surgeries. If combined with the needling distance, this sensor can also measure the hardness of tissues and provide tactile feedback for the robot to adjust the needling displacements and applied forces if different types of tissues are encountered during the operation.

### Monitoring the robot-assisted resection of tissues

Surgical operations, such as gynecological operations, include the uterus, ovary, bladder, ureter and other organ tissues, as well as nerves, blood vessels and lymph nodes of para-uterine peripheral tissues. During surgery, the resection process should be smooth and steady so that the cut tissue surface can be tidy without a rough texture, otherwise it will affect wound healing [48]. And if the resection path is not carefully planned, it is possible to cause accidental injury to the adjacent tissues, blood vessels or nerves, thus increasing the probability of postoperative complications [49]. In this section, we study the capability of the sensor unit to monitor the process of knifepoint resection in robot-assisted operations. Thanks to its ultra-high sensitivity, the sensor can be utilized to monitor the cutting process, and identify any interruption to the smooth advancement of the scalpel.

**Figure 6.**
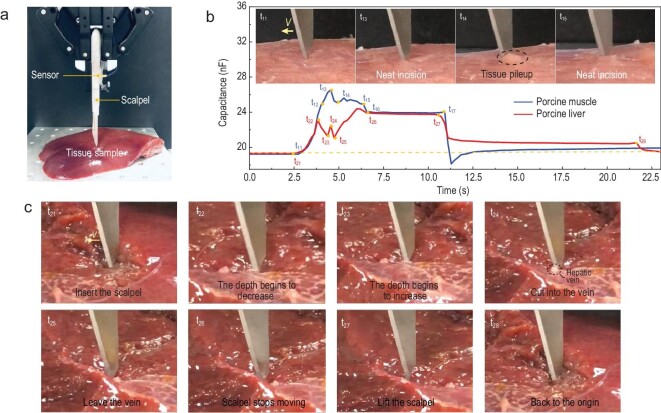
Monitoring of the surgical resection of tissues by MBM sensors. (a) The experimental schematic diagram. (b) The cutting forces monitored by MBM sensors during the resection of porcine muscle and liver. The insets are photographs of the cutting of porcine muscle at different times. (c) Photographs of the cutting of porcine liver tissues at different times as the scalpel advances.

As shown in Fig. 6a, the MBM sensor is mounted on a robotic gripper to demonstrate the process of tissue sample resection. Porcine tenderloin and liver are adopted as cutting samples due to the similarity of their mechanical properties to human muscles and inner organs, respectively [47,50]. A scalpel with a No.10 blade (used to cut tissues such as skin, subcutaneous tissue, muscle and periosteum) and No.3 handle, is used for the resection of porcine tenderloin and liver. First, we use the tactile sensor to monitor the neatness of incision during the cutting process of tenderloin tissues, as shown in Fig. 6b. Before t_11_, the robotic gripper holds the scalpel to approach and cut into the sample. Between t_11_ and t_13_, the capacitance of the tactile sensor increases rapidly from 19.24 nF to 24.80 nF. In this process, the robotic gripper speeds up and the tissue incision is relatively smooth and tidy, as seen in the inset of t_13_ in Fig. 6b. As the cutting depth is relatively large and the muscle tissues gradually pile up at the tip of the blade, this contributes to the increased cutting forces at t_13_ (Movie S4). Due to the sharpness of the blade, further advancement of the blade results in the incision of the pileup and there is a noticeable release of cutting force at t_14_. From t_15_, the robotic gripper slows down and the motion is stopped at t_16_. At t_17_, the scalpel is retracted from the muscle tissue. These observations indicate that, by monitoring the capacitance of the sensor, it is possible to identify resection status and distinguish interruption, such as the pileup of the tissue.

Porcine liver tissues are strain-rate sensitive with viscoelastic and super-elastic properties, as they contain complex tissues such as veins, arteries and bile ducts. We also monitor the resection of porcine liver tissue using the MBM tactile sensor. Although the experimental procedures of incision are similar to those of pork loin, different responses are obtained due to the differences in mechanical properties, surface roughness, incision wound fluid and exudates between porcine liver and pork loin. As shown in Fig. 6b, the scalpel begins to cut the liver as the robotic gripper starts moving after t_21_. From t_22_ to t_23_, due to the uneven surface of the liver, the depth of the blade into the liver is significantly reduced, and the capacitance is reduced by 1.88 nF. Due to the stickiness of the tissue, the tissue is stretched rather than incised during this process, and this ‘knife sliding’ phenomenon can be clearly seen in the movie (Movie S5). In t_23_– t_24_, with the scalpel moving on, the depth of the tip in the liver increases and capacitance rises. At t_24_ the blade cuts into a hepatic vein, which is softer than the liver tissue, and the cutting force declines (the inset of t_24_ in Fig. 6c and Movie S5). After t_25_, the cutting depth increases and the cutting force also increases. At t_27_, the scalpel is retracted from the tissue and part of the tissue is still in contact with the tip of the scalpel. After lifting the scalpel at t_28_, the robotic gripper moves to the initial position, and the capacitance value of the sensor also returns to the initial value. This experiment confirms that the MBM sensor can monitor variation in cutting depth and the presence of other materials/tissues during the resections of rather soft inner organs.

## CONCLUSION

In summary, we have proposed bio-inspired capacitive ionic e-skin sensors for the monitoring of delicate operations by surgical robots. Based on the surface microstructure of *M. aquaticum* pollen, the structure of a bionic sensitization system was designed, and the existing biological model was optimized in combination with the characteristics of functional materials. The resultant multilevel bionic microstructures endow the tactile sensor with an unprecedented high pressure measurement sensitivity of 9484.3 kPa^−1^ (<15 kPa). Because of its inherent flexibility, the capacitive ionic skin sensor can be conveniently attached to robotic manipulators, so as to realize the delicate operations of robotic needle threading, as well as the tactile monitoring of needle insertion and resection in robot-assisted invasive surgery. During mass production of the devices, in order to improve the consistency of sensor performance, it is necessary to strictly define preparation processes and conditions. In addition, the electrode will be oxidized after long-term use, and the oxidation of the electrode can be slowed down by improving the packaging process. Due to the facile fabrication schemes and high sensitivity, ionic e-skin sensors also have great application prospects in robot dexterity operations, artificial limbs, human–machine interfaces and so on.

## METHODS

### Preparation of the ionic gel dielectric layer with MBM

Ionic gel mixed solution consists of organic polymer compound P(VDF-HFP) (Sigma–Aldrich, P304908) and ionic liquid [EMIM] [TFSI] (Aladdin, E101506). P(VDF-HFP) pellets (1.5 g) were added into a reagent bottle filled with acetone, and the mass ratio of P(VDF-HFP) pellets to acetone was 10 : 1. A magnetic bead was placed in the reagent bottle and stirred at room temperature with a magnetic stirrer for 6 h until P(VDF-HFP) was completely dissolved. In the prepared P(VDF-HFP) solution, according to the mass ratio of P(VDF-HFP) pellets to [EMIM] [TFSI], the corresponding weight of [EMIM] [TFSI] was dripped with a rubber head dropper, and the mixture was continuously stirred for 30 min to form an ionic gel solution. The mass ratio of P(VDF-HFP) pellets to [EMIM] [TFSI] was 1 : 3.5, if not specified otherwise. A fresh *C. zebrine* leaf was cleaned with deionized water, and slowly blow-dried with nitrogen. After removal of the main vein from which the leaves were removed, and the irregular edges, the middle portion of the leaves was taken out to be cut into a rectangle with a size of 25 mm × 75 mm and attached to a glass slide by 3M tape. Next, the entire slide was fixed to the bottom of the customized glasswork with a depth of 2 mm. PDMS (Dow Corning Sylgard 184) was poured into an acrylic box and spread to the same height as the acrylic, and placed on a horizontal workbench for 48 h to closely fit the fluidity of PDMS with the blade microstructure. The sample cured naturally, then was peeled off and attached to the customized glass template frame with 3M double-sided adhesive tape. The prepared ionic gel solution was dripped into the glass template frame with a rubber dropper, and then placed in an environment of 26–40°C and 60%RH–99%RH for 3 h, and then naturally cured. Due to the shrinkage of ionic gel, it was easy to peel. The prepared ionic gel was peeled off and laser cut into 15 mm × 15 mm squares.

### Characterizations and measurements

The microstructure of the dielectric surface, and the surface morphology of the leaf template, were observed by SEM (Hitachi SU3900). A laser scanning confocal microscope system (VK-X200K, Keyence) was used to obtain the three-dimensional surface morphology of the dielectric layer and the cross-sectional dimensions of the dielectric layer. The statistical distribution of the micro-cones and micro-pits of the dielectric layer was obtained by software (ImageJ). If not specified otherwise, the capacitance signal of the sensor was measured by LCR meter (E4980AL, KEYSIGHT) at a testing frequency of 1 kHz, and displayed and recorded in real time by LabVIEW (National Instruments, USA). A motorized Z-stage was used in combination with a force gauge (HP–5N or HP-50N by HANDPAI) to apply a well-defined pressure during the measurements. The sensor was placed on a precision moving platform (FlexTest Mini-S2-P, Hunan NanoUp Electronics Technology Co., Ltd) for a cyclic bending test at a constant speed of 5 mm/s.

## Supplementary Material

nwac227_Supplemental_Files
