# Supplementary material for: Monitoring the delicate operations of surgical robots via ultra-sensitive ionic electronic skin
Source: Natl Sci Rev. 2022 Oct 21;9(12):nwac227. doi: 10.1093/nsr/nwac227 (PMC9798889; doi:10.1093/nsr/nwac227)
Supplement: nwac227_Supplemental_Files [file nwac227_supplemental_files.zip › Supplementary_data.pdf]

Supplementary Information for

Monitoring Delicate Operations of Surgical Robots by Ultra-sensitive Ionic Electronic Skin

Danyang Wei[1], *Jiajie Guo*[1], *Yuqi Qiu*[1], *Shaoyu Liu*[1], *Jiangyan Mao*[1], *Yutian Liu*[2], *Zhenbing Chen*[2], *Hao Wu*[1]*, *Zhouping Yin*[1]*

D. Wei, Prof. J. Guo, Y. Qiu, S. Liu, J Mao. Dr. Y. Liu, Dr. Z. Chen, Prof. H. Wu, Prof. Z. Yin

[1]State Key Laboratory of Digital Manufacturing Equipment and Technology, School of Mechanical Science and Engineering, Huazhong University of Science and Technology, Wuhan, Hubei, 430074, China.

[2]Department of Hand Surgery, Union Hospital, Tongji Medical College, Huazhong University of Science and Technology, Wuhan, Hubei, 430022, China.

*Correspondence authors. E-mails: hwu16@hust.edu.cn, yinzhp@hust.edu.cn,

This PDF file includes:

Supplementary text

Figure S1 to S18

Tables S1

SI References 1 to 18

Other Supplementary Information for this manuscript include the following:

Movie S1 to S5

Supplementary text

An overview of electrical double layer (EDL) components.

When two conductive phases (such as an electrode and an electrolyte) are in contact, their Fermi levels reach equilibrium and a potential is formed at the interface[1]. Depending on this potential and the composition of the solution, one phase becomes negatively charged and the other one becomes positively charged when an electrical double-layer is developed at the electrode-solution interface. In the first model proposed by Helmholtz for the structure of the electrical double-layer, it is considered that counter-charges in the solution locate at a molecular order distance from excess charges on the electrode surface. Such a structure resembles a parallel-plate capacitor, whose capacitance is described by:

$$C_{EDL} = \frac{\varepsilon\varepsilon_0 A}{d} \quad \textbf{(1)}$$

where A is the surface area of the electrode, d is the distance between the two sheets of charges, ε and ε_0 refer to the dielectric constant of the medium and the permittivity of free space, respectively. As apparent by this equation, the Helmholtz model predicts a constant capacitance for the double-layer. However, experimental results show that it changes with potential and concentration. Accordingly, either ε or d or both should depend on potential and concentration. As a unique characteristic of the ionic-electronic contact, the interfacial capacitance lies on the formation of an EDL, in which electrons on the electrode and the counter ions from the iontronic film accumulate and attract each other at a nanoscopic distance (d is at the nanoscale), which leads to an ultrahigh unit-area capacitance[2].

The Gouy–Chapman–Stern (GCS) model

The standard model to describe the equilibrium EDL structure at the junction metal/electrolyte (dilute solutions) is the GCS mean-field model which can quantitatively explain most experimental results. It subdivides the EDL into (i) a first sheet of uniform constant electronic charges on the metal surface, (ii) a charge-free inner compact layer or Helmholtz layer (also known as Stern layer) of a few angstrom in thickness and constant charge, and (iii) an outer, semi-infinite diffuse layer (also known as Gouy Chapman layer) that extends into the bulk electrolyte and contains anions and cations of a certain distribution[3].

In the diffuse layer part of the GCS's model, the electrolyte is treated using the mean-field PB model. For dilute solutions with negligible correlations, the ion concentrations at a perpendicular distance x≥0 from a large lateral surface area metal electrode (one-dimensional problem in which the edge effects are ignored) are assumed to be related to the mean electrostatic potential ψ_x by the continuous Boltzmann distribution function[4]:

$$C_{x_i} = C_{x_i}^0 \exp\left(-\frac{Z_i F \psi_x}{RT}\right) \quad \textbf{(2)}$$

where $C_{x_i}^0$ and Z_i are the average ion concentration (for ψ_x = 0) and charge number of species i (cation and anion), respectively. The term $RT/F = k_BT/e$ (with R the ideal gas constant, F the Faraday's constant, k_B the Boltzmann s constant, e the elemental electronic charge, and T the absolute temperature) is the thermal voltage.

The total net charge per unit surface Q_x within the diffuse layer as:

$$\left(\frac{d\psi_x}{dx}\right)_{x=d} = -\frac{Q_x}{\epsilon} \quad \textbf{(3)}$$

where ϵ is the uniform dielectric permittivity of the solvent in units of the vacuum dielectric constant ϵ_0.

The total net charge within the diffuse layer as:

$$Q_x = \sqrt{8\epsilon RT C_x^0} \sinh\left(\frac{zF\psi_d}{2RT}\right) \quad \textbf{(4)}$$

To determine the specific capacitance of the diffuse layer, the net charge (Eq. (4)) is differentiated with respect to the potential, which gives the well-known nonlinear expression:

$$C_{\text{diff}} = \sqrt{\frac{2\epsilon z^2 F^2 C_x^0}{RT}} \cosh\left(\frac{zF\psi_d}{2RT}\right) \quad \textbf{(5)}$$

The overall EDL capacitance of the electrode is then computed from the combination in series of the two capacitances arising from the Helmholtz inner layer (of constant capacitance $C_{\text{H}} = \epsilon/4\pi d$ with d being its thickness) and the diffuse layer, such that:

$$C_{EDL}^{-1} = C_H^{-1} + C_{\text{diff}}^{-1} \quad \textbf{(6)}$$

Overall capacitance analysis of MBM iontronic pressure sensor.

According to Gouy–Chapman–Stern model, this interface without electrochemical activation can be simply modelled as a capacitive element, and the equivalent circuit of the whole device is shown in Figure S4. The total capacitance *C* is obtained by the following formula:

$$C^{-1} = C_{bottom}^{-1} + C_{\mathrm{top}}^{-1} \quad \textbf{(7)}$$

where C_{top} and C_{bottom} represent the capacity of the top and bottom interfaces, respectively. The bottom contact area is much larger than the top contact area, thus C_{bottom} is typically much larger than C_{top}, hence the term C_{bottom}^{-1} contributes little to the total capacitance and can be ignored. Therefore, the sensitivity of the device is largely contributed by the rough surface. Here, the top EDL capacitances are in parallel to form one top EDL capacitance，

$$C_{top} = C_{air} + \sum C_{EDL-i} \quad \textbf{(8)}$$

When there is no external force load, the top dielectric-electrode contact area is very small, thus $C_{top}(\approx C_0)$ is mainly determined by C_{air}. With the increase of external load, the change of dielectric layer thickness can be neglected. C_{top} is mainly determined by the number of cones contacting the top electrode layer and the contact area of each cone.

Finite Element Analysis (FEA).

COMSOL (6.14) was used to carry out three-dimensional model and numerical analysis. The ECC was embedded in PDMS with similar mechanical properties to PDMS, in order to save computational resources and time, the ECC layer was ignored and the model was simplified as a square film with a size of 100 μ m × 100 μ m. The density of PDMS[5] and ionic gel[6] are about 1 g/cm^3 and 1.1 g/cm^3, respectively. The Poisson's ratio of PDMS and ionic gel are about 0.49 and 0.42 [7-8]. The young's modulus of PDMS[7] is 1.365 MPa. Ogden material model was used to describe the non-linear stress-strain behavior (Figure S11) of ionic gel[6]. The 80μm micro-pit structure was located in the center of the square film, and the height of the micro-cone was approximately subject to normal distribution with size between 22 and 30 μm, according to our observation by SEM.

We found that in the four stages of loading, there were different stress distributions: in the initial state, as shown in Figure 2d (i), only the cone with the highest surface is in contact with the electrode layer, and the micro cones in the micro-pit structure have no contact with the electrode layer; At 0-15 kPa, as shown in Figure. 2d (ii), all the micro-cones in this part are subjected to certain stress and strain at the same time. With the increase of strain, the effective contact area between the dielectric layer and the electrode layer increases. With the increase of external load, the micro-cones on the spherical surface of the micro-pit structure are not completely perpendicular to the upper electrode layer, but contact at a certain angle. Under the action of external force, individual cones in the micro-pit structure, certain bending deformation begins, which renders the contact areas of the dielectric layer with only a small amount of cone contact on the surface greatly increase. With the further increase of external load, under the pressure of 15 kPa-35 kPa, the local pressure is concentrated at the cone tip, and the stress and strain are further increased. The micro-cone structure that is not in the pit is not flattened, but the area is still increasing. With the increase of external load, the number of cones with bending deformation in the middle micro-pit structure also increases, thus increasing the contact area. Under the pressure of 35 kPa-85 kPa, the stress distribution of the surface cone increases, and the strain also increases obviously. With the increase of external pressure, the bending degree of the cone in the middle micro-pit structure increases, the contact area also increases, and the thickness of the dielectric layer begins to decrease slightly, which contributes to the improvement of the pressure sensitivity, and is also the reason why the sensor can maintain a high sensitivity in the medium-voltage region. Under the pressure of 85 kPa-155 kPa, the contact area of the cone on the surface begins to increase slowly, the bending degree of the cone in the middle pit structure further increases, and the thickness of the dielectric layer decreases. The change of the capacitance of this part of the device is mainly determined by the change of the thickness of the dielectric layer and the contact areas between the cone on the surface and the electrode layer. When the external load exceeds 155 kPa, the change of the capacitance of the device is mainly determined by the change of the thickness of the dielectric layer, which is also the reason why the sensor can maintain a certain sensitivity in the high voltage range.

Preparation of the flexible electrodes and the sensor

Based on our previous work, the ECC was prepared by mixing treated silver flakes and PDMS at a mass ratio of 3:1 and being added to a mortar and mixed for 5 min. First, 10% poly(vinyl alcohol) (PVA) aqueous solution was spin coated onto a clean glass slide as the sacrificial layer, and then dried at 80 °C for 20 min. The ECC was then patterned on the PVA layer by stencil printing. The stencil mask was made of a 100 μm thick poly(vinyl chloride) (PVC) tape and patterned by a CO_2 laser cutting system (HZZ-V300, HZZ Laser). A screen-printing scraper was used to evenly smear and scrape the ECC materials on the PVC mask. Then the PVC mask was removed. After thermally curing ECC at 160 ℃, spin-coating and curing of a thin layer of PDMS to cover the ECC pattern. Subsequently, the PVA sacrificial layer was dissolved in 80 ℃ water bath to peel the sample from the glass slide. The total thickness of the electrode was about 100 μm.

The flat surface of the MBM was placed on the surface of the ECC/PDMS lower electrode. Since both MBM and the underlying PDMS of the lower electrode were sticky, a gentle press allowed both to be attached tightly without bubbles. Then that ECC/PDMS upper electrode was placed on the MBM and aligned with the face-to-face orthogonal lower electrode. Due to the residual PVA on the alignment side, which had a large viscosity, the device could be packaged well at the edge with 3M tape. The sensing area of the sensor is a circle with a diameter of 8mm, and the size of the whole sensor is 25mm×25mm, as shown in Figure S3. It is noted that the measurment performance of the device samples prepared in the same process is very stable (Figure S15).

Fabrication of sensor and sensor array

The upper electrode layer and the lower electrode layer are composed of 12 capacitor units in a 3×4 arrangement, as shown in Figure 4e. Each capacitor unit has a diameter of 0.8mm and a connection line width of 0.2mm.

Experiments in robotic operations

A robotic gripper (2FINGER-85, ROBOTIQ) was mounted on a commercial robotic arm (UR5, UNIVERSAL ROBOTS) and all used MBM sensor units were 8mm in diameter. As shown in the threading task, a needle (pinhole: ~0.7mm in width and 3.6mm in length) with a thickness of 0.9mm was fixed on acrylic which had a hole with a width of ~0.6mm and a length of 3.2mm. The soft suture (diameter of 0.1 mm) held in the robotic gripper

was remotely controlled to push through the eye of the fine needle by reading the input signal from the tactile sensor. For monitoring tissue resection, the MBM sensor was compared with the commercial sensor (FlexiForce A201 Sensor, Tekscan). In order to keep the freshness of organs and tissues, normal saline was regularly sprayed (see Supplementary text for details).

Sample preparations.

The porcine livers used in the experiment were all taken from live pigs, which were cut out after slaughter, and sent to the laboratory about 3 hours later. In order to keep the moisture of organs and tissues, physiological saline solution was sprayed regularly after being sent to the laboratory, during the preparation of samples and before the experiment. The average density of porcine liver samples was ~1056 kg/m^3. In order to minimize the influence of postmortem time on the mechanical properties of tissues, each experiment was controlled to be conducted within 8 hours after death. And porcine loin was treated in a similar way.

Figures

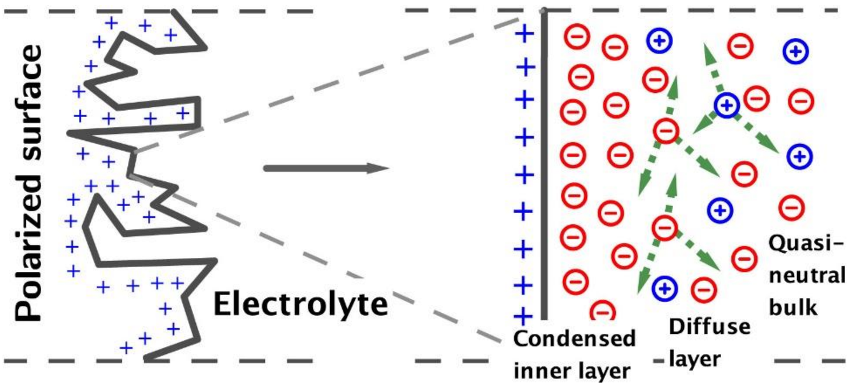

Figure S1. Schematic representation of electric double-layer structure at a positively-polarized blocking electrode/electrolyte interface [4]. Its thickness (a measure of its capacity) is usually in the order of a few nanometers at most.

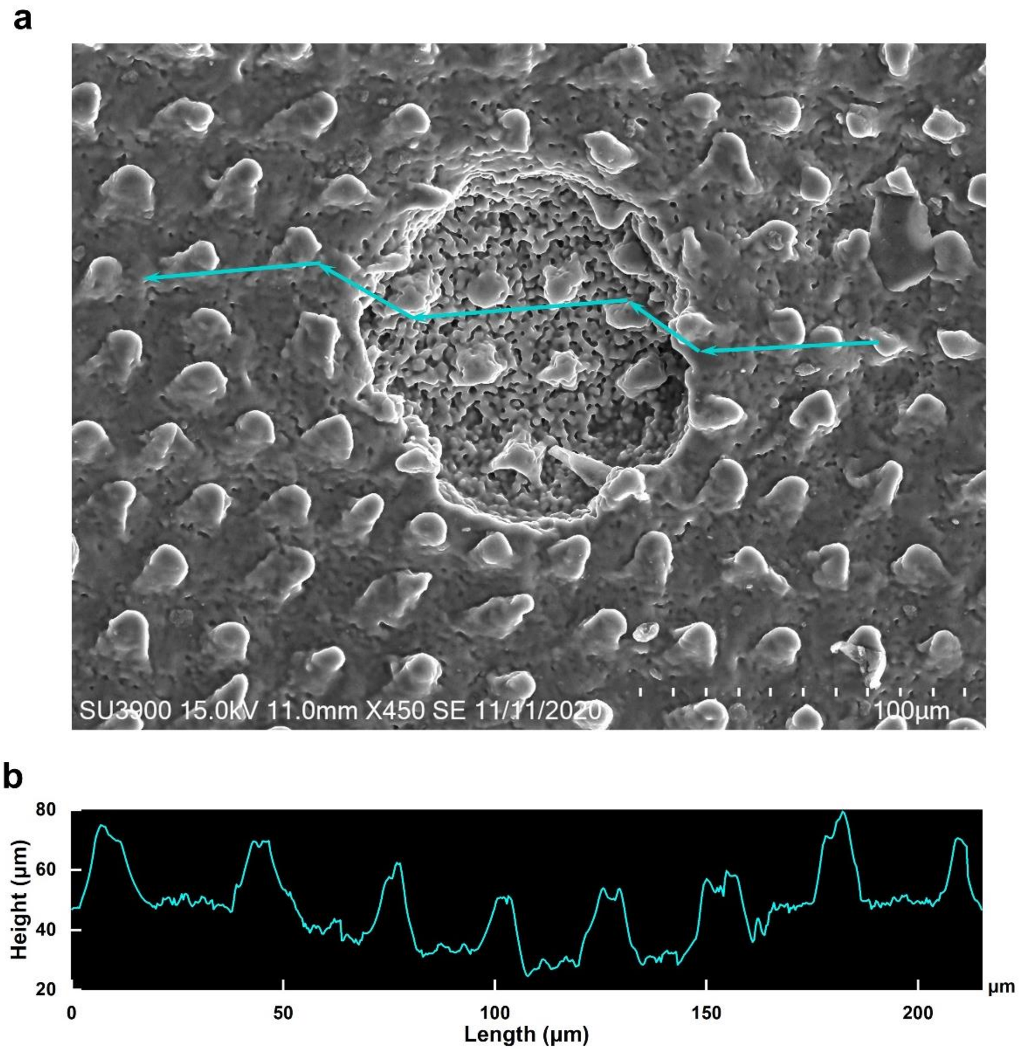

Figure S2. characterizations of MBM-based ionic gel dielectric film. (a) SEM image of the MBM-based ionic gel dielectric film. **(b)** Cross-sectional measurement at the site of the blue line in **(a)**, and the measurement direction followed the arrow. It can be seen that the depth of micro pits is ~ 31.8 μm and the distribution of micro cone structures in micro pits.

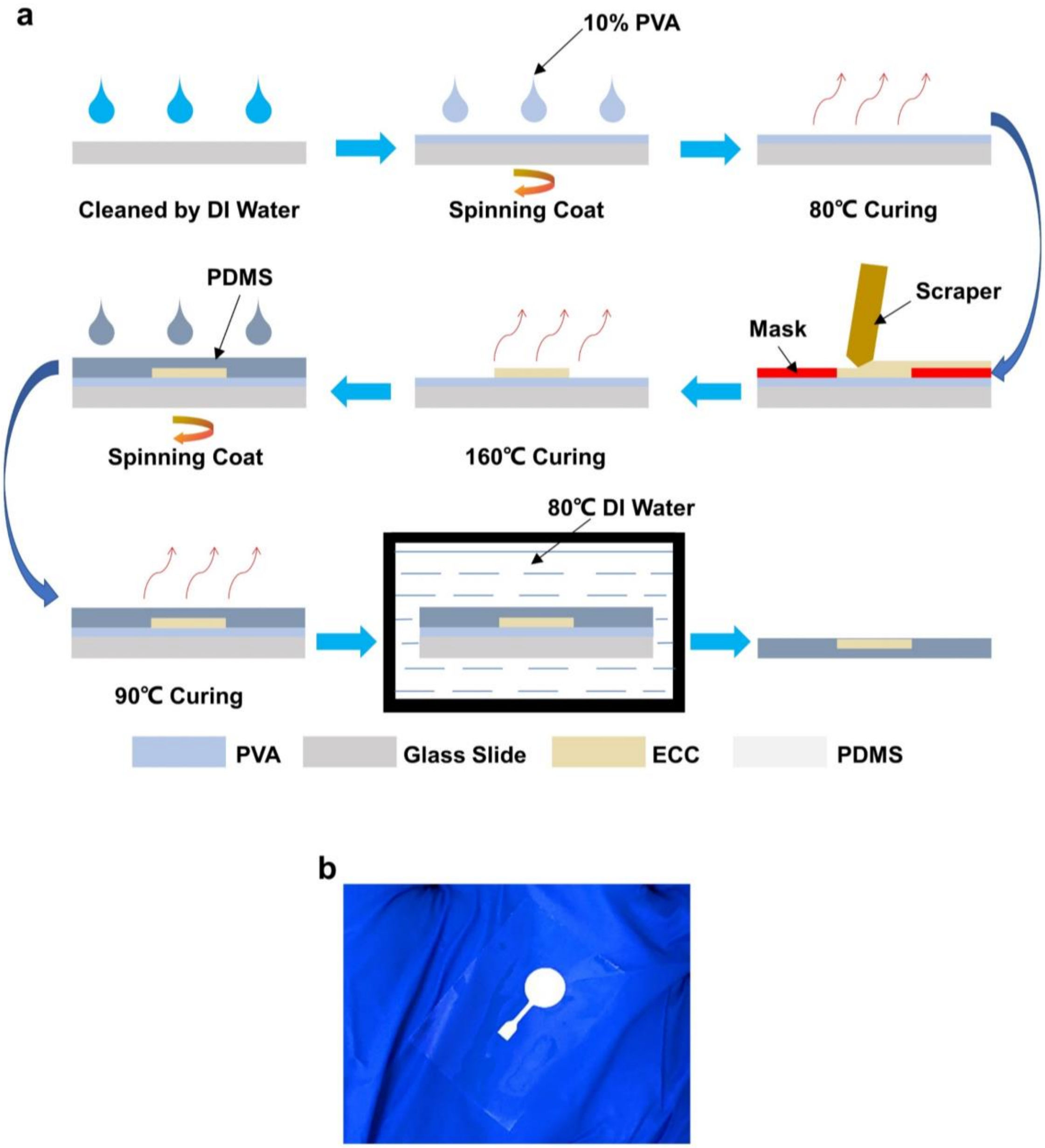

Figure S3. Schematic illustration of the detailed fabrication processes of the ECC electrode layers. (a) As a sacrificial layer, the PVA was spin-coated onto a clean glass slide. The ECC was patterned by a patterned PVC mask and covered by a thin layer of adhesive PDMS. The electrode layer was finally peeled off by heating in a water bath. **(b)** Image of the ECC electrode after peeling off.

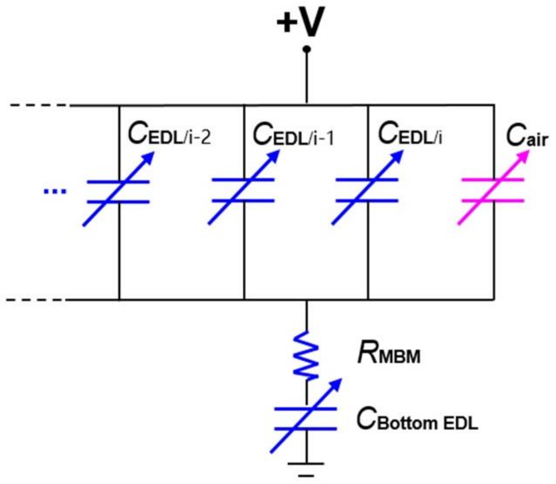

Figure S4. The equivalent circuit model of the MBM e-skin sensor.

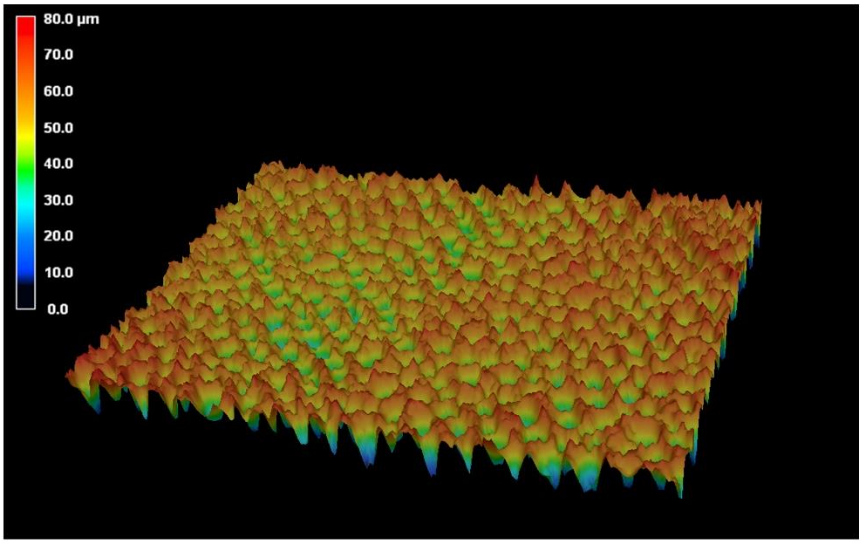

Figure S5. 3D topology of biomimetic PDMS template after microstructures of the *Calathea zebrine* leaf.

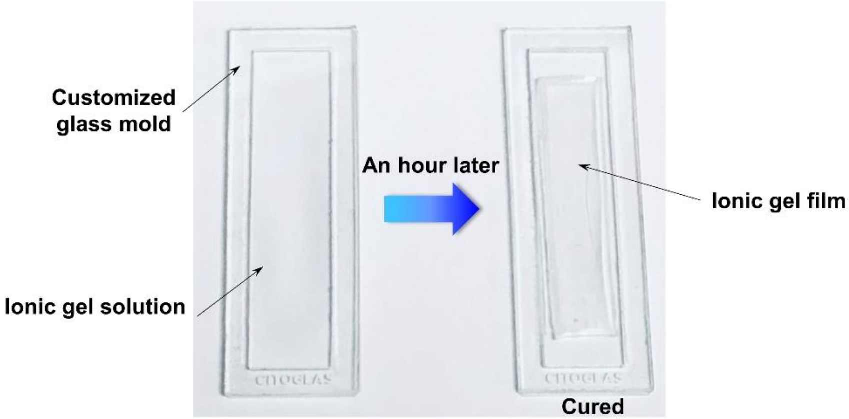

Figure S6. The shrinkage of the ionic gel after curing. The blended solution of ionic gel is based on P(VDF-HFP) elastomer and [EMIM] [TFSI] ionic liquid, and acetone as solvent. The groove was made by attaching a rectangular acrylic frame to a clean glass slide. Filling the groove with the ionic gel mixture (left), then let it naturally cure for one hour. Compared with the volume before curing, the ionic gel film shrank by about 75% (right). This was due to the fact that acetone volatilized during curing.

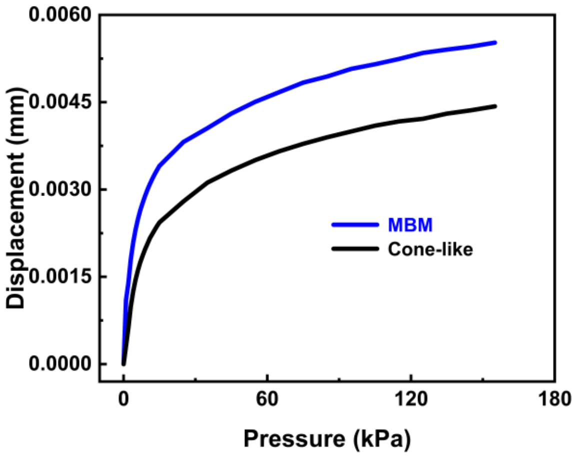

Figure S7. Finite element simulation results of load - displacement behavior of sensors with different dielectric surface structures. Under the pressure of 0-155kPa, the distance between the upper and lower electrodes of dielectric layers with different structures changes. MBM refers to the sensor with multi-stage microstructure while Cone-like reference to the sensor with only micro-cone microstructure.

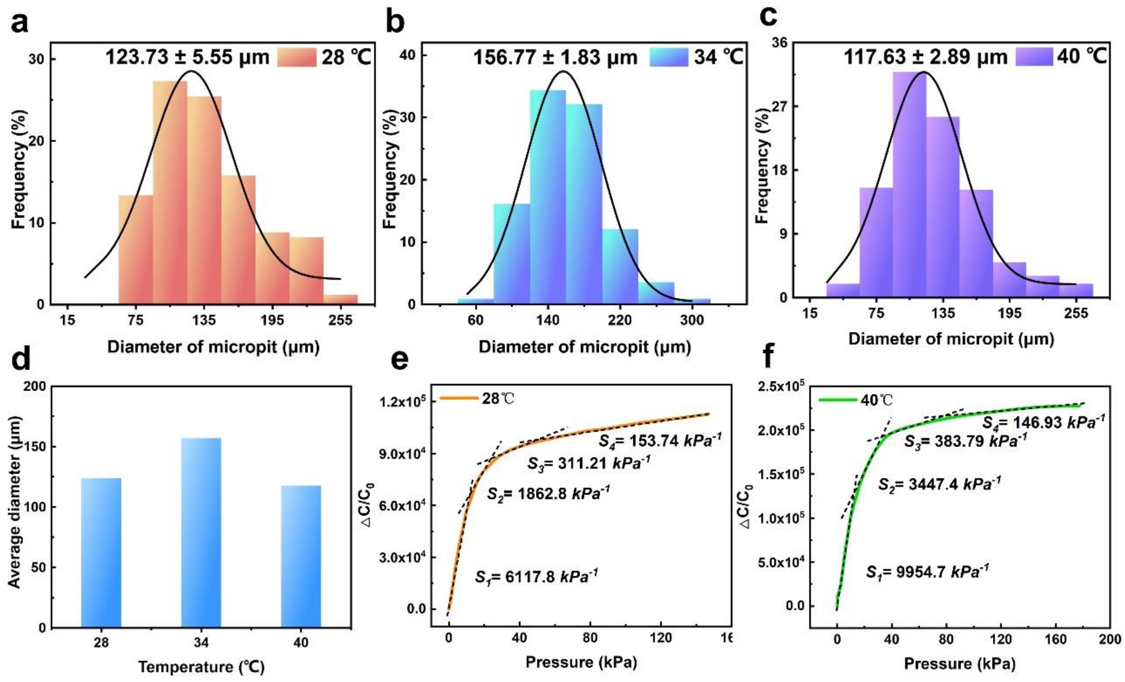

Figure S8. Characterizations of surface microstructures and sensing performance characterizations of MBM-based dielectric layers at different curing temperatures. Diameter distribution of micro-pit structures on ionic gel films, when the curing temperature is **(a)** 28℃, **(b)** 34℃ and **(c)** 40℃. All of the ionic gel films used the same microstructure PDMS template. **(d)** Study on average diameter of micro-pit structures caused by curing temperature. With increasing temperature, the average diameter of micro-pit structures has the tendency of increasing first and then decreasing. The relationship between the change of relative capacitance and the pressure applied to the MBM e-skin sensor using ion gel films prepared at **(e)** 28℃ and **(f)** 40℃. Obviously, the range of capacitance/pressure change of the sensor has four stages, which is consistent with previous FEA results (Figure 2d).

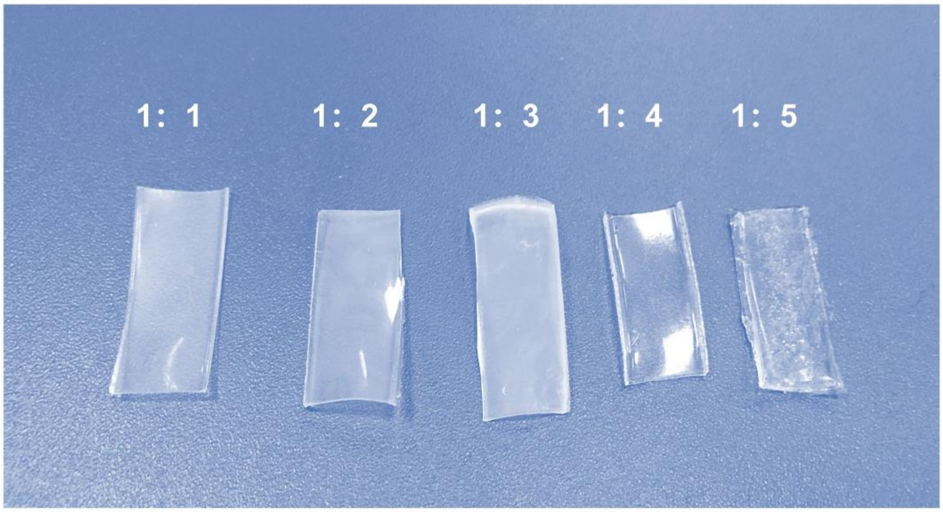

Figure S9. Photographs of ionic gel films with different ratio of P(VDF-HFP) to [EMIM][TFSI]. From left to right, the composite ratio of P(VDF-HFP) and [EMIM][TFSI] ranges from 1: 1 to 1: 5, with a constant film thickness of 120 μm. It can be seen that with the increase of the proportion of [EMIM][TFSI], the transparency of the films increases. However, the ionic gel film with the ratio of 1: 5 has a rough surface, while the other ionic gel films have a smooth surface. The surface roughness is possibly due to the large proportion of ionic liquids, which renders it difficult for P(VDF-HFP) pellets to completely dissolve in acetone. Even though the unit-area capacitance of the dielectric film with the weight ratio of 1: 5 is higher than that of other films (Figure 3a), this indicates that the ratio of 1:5 isn't suitable for the preparation of surface microstructure.

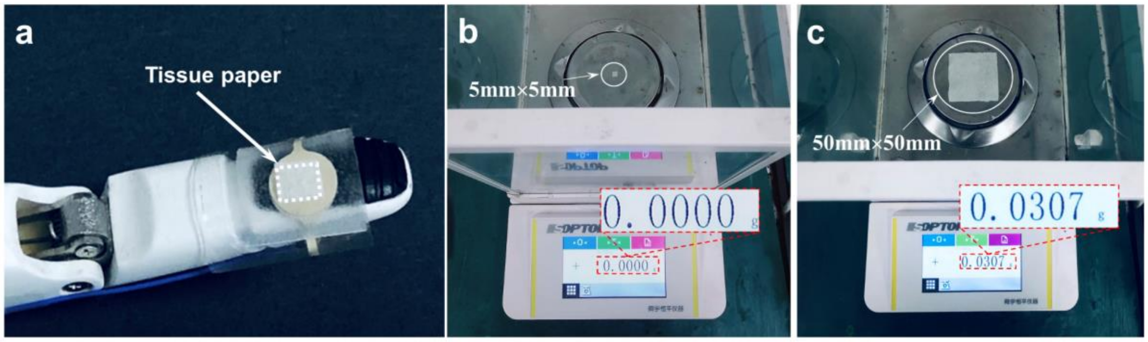

Figure S10. Demonstrations of the detection limit by means of loading a piece of tissue paper with weight of ~0.307 mg. (a) A photograph of 5mm × 5mm tissue paper placed on the sensor, which is integrated in the bionic hand. **(b)** The weighing of the tissue paper in (a) by a precision electronic scale shown in the picture. It can be seen that the precision scale (AE 223，Shanghai Yu Shun Hengping Scientific Instrument Co., Ltd.) can't detect the piece of tissue paper, but our sensor can detect the tiny pressure. (Figure 4c). **(c)** The weighing of a 50mm × 50mm tissue paper by the precision electronic scale. Hence, it can be calculated that the weight of a 5mm × 5mm paper is ~0.307mg. The tissue paper (purchased from Vinda) is composed of three layers, only one layer was used for the tests.

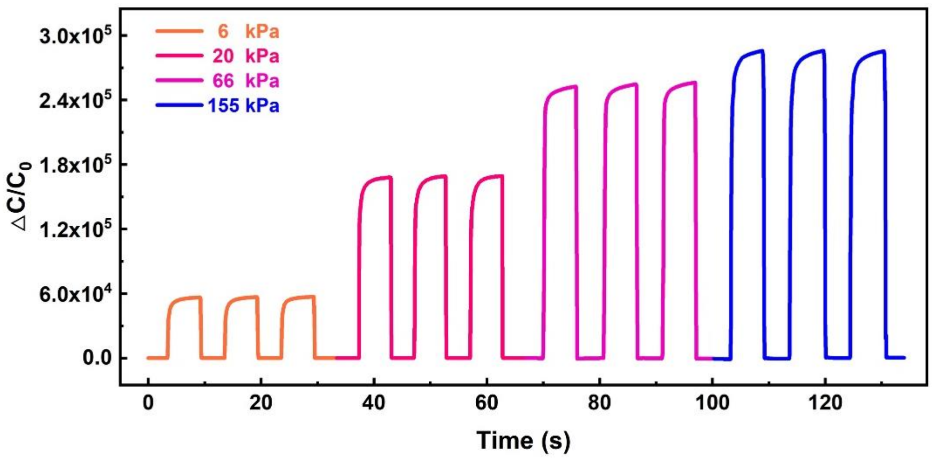

Figure S11. Relative capacitance changes of the sensor with the gradual increase of applied pressure.

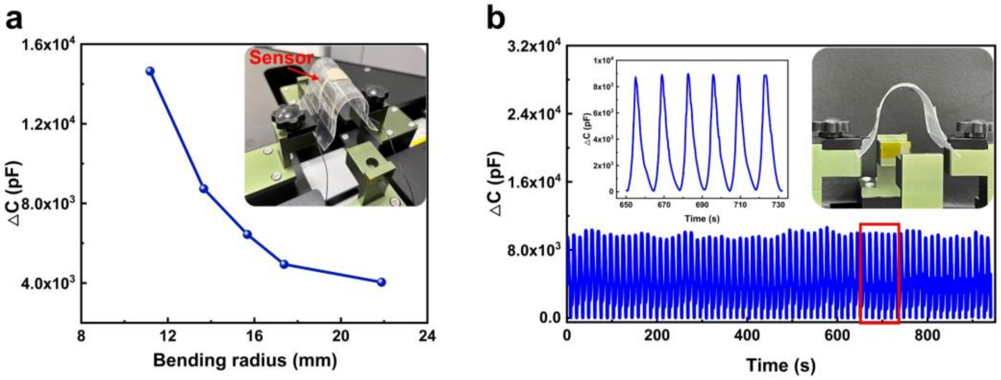

Figure S12. Sensing properties of the MBM-based ionic e-skin sensor under bending. (a) Capacitance response as a function of bending radius. (b) Cyclic dynamic bending sensing with bending radius of 13.66 mm. Bending caused in-plane tensile and compressive stresses on top and bottom side of the neutral axis, respectively. Meanwhile, with the decrease of bending radius, the normal pressure increased, which caused the sensor capacitance to increase.

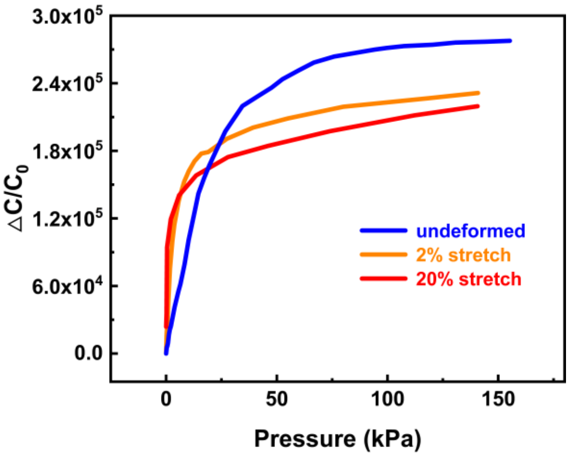

Figure S13. Response of MBM-based sensor to pressure in undeformed and stretched status. When the pressure was within the range of 0–10 kPa, the sensitivity of the sensor under tension was relatively high. This was because tension applied a certain normal pressure to the sensor, and due to the external pressure, the response increased. As stretching caused the middle dielectric layer of the sensor to become thinner, the sensitivity of the stretched sensor decreased, compared to the unstretched device at pressure above 15kPa.

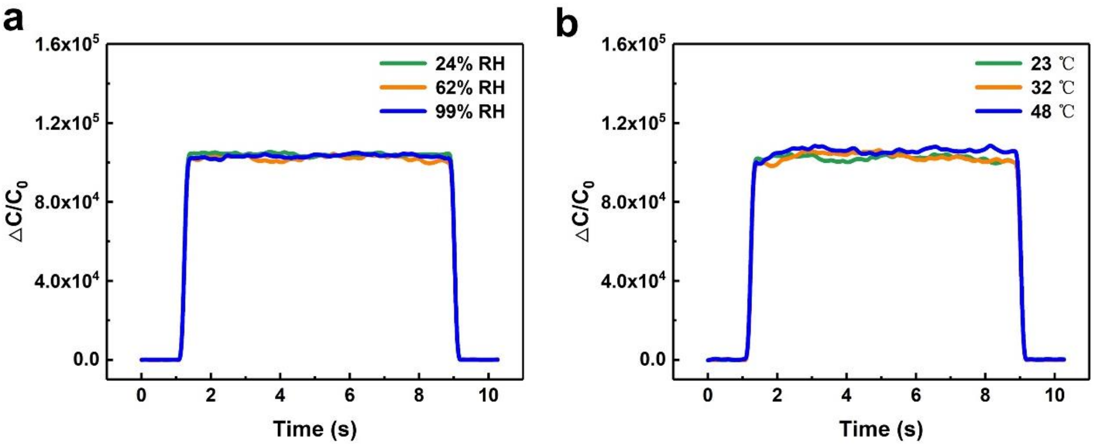

Figure S14. Effect of ambient temperature and humidity on sensor performances. The sensing performances (at 10 kPa) of the MBM-based sensor under **(a)** different humilities and **(b)** different temperatures.

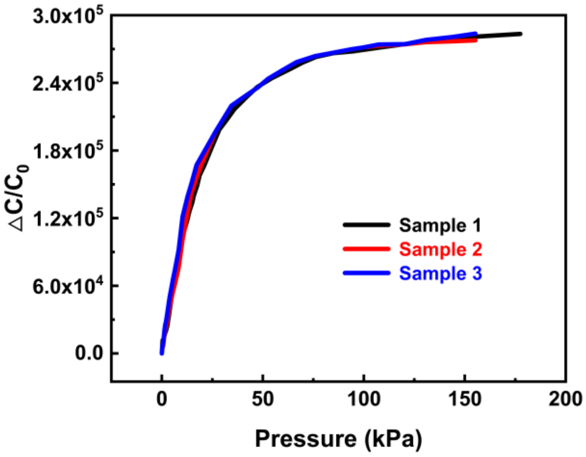

Figure S15. Repetitive experiments on measurment performance of the MBM-based ionic e-skin sensors prepared under the same preparation conditions in different batches.

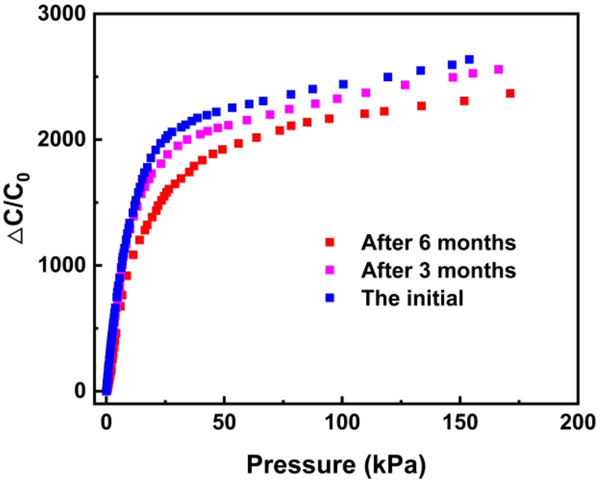

Figure S16. Measurement performances of the same device at initial, 3-month and 6-month intervals. Testing frequency was 300 kHz.

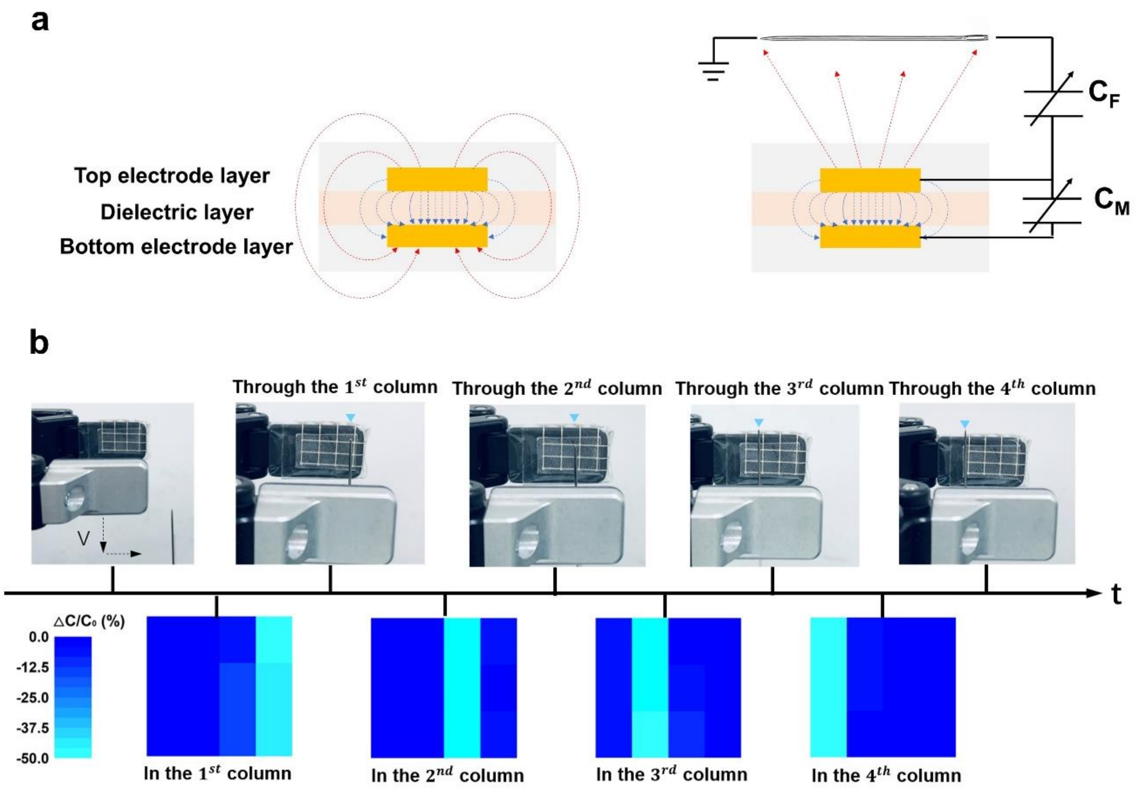

Figure S17. Working mechanism and applications of proximity sensing. (a) Equivalent electrical circuit of the proximity sensing. For each capacitive unit in the sensor array, the overall capacitance of the sensor is the combination of the fringe capacitance C_F and the coupled capacitance between the top and bottom electrodes C_M. When a conductive object approaches the device, it acts as a third electrode which capacitively coupled to the sensor element, reducing the coupling between the two electrodes C_M. Thus, the cross-grid array can be used as proximity sensors. **(b)** Motion tracking of a surgical needle through the sensor array, the arrays are scanned to identify the needle's locations, when the robotic manipulator moves. The relative capacitance variations of the sensor array are shown (bottom) as the needle passed through each column of the sensor array in sequence (top).

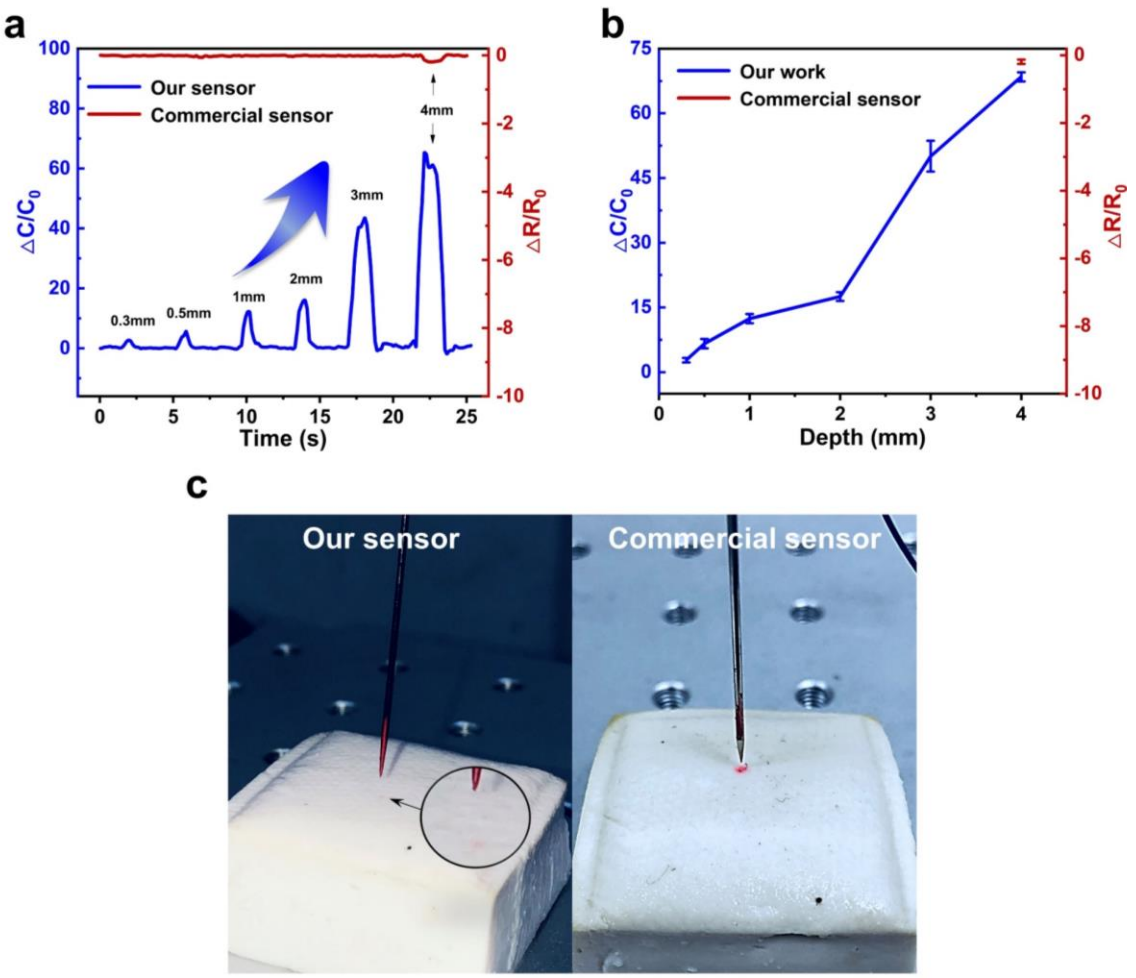

Figure S18. Needle penetration perception of ultra-tender bean curd by the sensors. (a) Real-time monitoring of capacitance variations of the sensor installed at the bottom of the needle, caused by the needle tip insertion into the tender bean curd. **(b)** The relationship between the sensors output and the distance between needle tip and tender bean curd. **(c)** The sensors detect the minimum distance between the bean curd and the needle tip, and the marks left by the needle tip on the surface of the bean curd. From the mark left by the needle tip, our sensor (left) can sense the very low penetration depth in bean curd, at which the needle doesn’t damage the bean curd. In contrast, at the minimum distance sensed by the commercial sensor (right), the needle already left significant and visible-pierced mark.

Table S1. The comparisons of the characteristics of capacitive pressure sensors reported in the literature and the sensor present in the current work.

Structure	Materials	Sensitivity [kPa^{-1}] (Range)	Minimum pressure	Response time	Applications related to robots	Ref
Micropatterned pyramidal	Ion gel and ITO	41 (0-400 Pa)<br>13 (0.5-5 kPa)<br>2 (5-50 kPa)	20 mg	20 ms	No	[8]
Double-sided sandpaper microstructure	Ion gel and CNT/PDMS nanocomposite	9.55 (0-0.2 kPa)<br>0.87 (0.2-2 kPa)<br>0.24 (2-8 kPa)	5 Pa	43 ms	No	[9]
Sandpaper microstructure	Ion gel and AgNWs	131.5 (0-1.5 kPa)<br>11.73 (5-27.7 kPa)	34.5 mg/1.12 Pa	43 ms	No	[10]
microcones	Ion gel and AgNWs	54.3 (0-0.5 kPa)<br>30.11 (0.5-10 kPa)<br>8.42 (10-40 kPa)<br>1.03 (40-115 kPa)	0.1 Pa	29 ms	Yes	[6]
Sandpaper microstructure	H_3PO_4 and PVA	3302.9 (0-10 kPa)<br>671.7 (10-100 kPa)<br>229.9 (100-360 kPa)	0.08 Pa	9 ms	No	[11]
Porous	BNF@PDMS and ITO	0.854 (0-0.5 kPa)<br>0.29 (0.55-2.1 kPa)	1 Pa	——	No	[12]
Natural eggshell inner membrane	PDMS and MXene($Ti_3C_2T_x$)/ AgNWs	1.5 (0-300 kPa)<br>0.5 (300-600 kPa)	16 mg	50 ms	No	[13]
Spacer	Ionic hydrogel and conductive fabric	0.24 (0-70 kPa)<br>1.5 (70-150 kPa)<br>0.13 (150-330 kPa)	35 Pa	18 ms	Yes	[14]
Multilayer double-sided sandpaper microstructure	Ion gel and Au	9.17 (0-2063 kPa)	13 Pa	5 ms	No	[15]
Porous	Ion gel and ITO	152.8 (0-20 kPa)	100 Pa	——	No	[16]
Porous nanocomposite	Conductive porous nanocomposite and Au	3.13 (0-1 kPa)<br>1.65 (1-5 kPa)<br>1.16 (5-10 kPa)<br>0.68 (10-30 kPa)	0.7 mg	94 ms	No	[17]
Pyramid microstructure and spacer	Ion gel and AgNWs	4.5 (0-1 kPa)<br>2.0 (1-10 kPa)	0.2 Pa	50 ms	No	[18]
**MBM**	**Ion gel and ECC**	**9484.3 (0-15 kPa)<br>3895 (15-35 kPa)<br>971.26 (35-85 kPa)<br>237.85 (85-155 kPa)**	**0.3mg/0.12Pa**	**24 ms**	**Yes**	**Our work**

REFERENCES

1. Azimzadeh Sani M, Pavlopoulos NG, Pezzotti S *et al*. Unexpectedly High Capacitance of the Metal Nanoparticle/Water Interface: Molecular‐Level Insights into the Electrical Double Layer. *Angewandte Chemie International Edition* 2022; **61**: e202112679.
2. Nie B, Li R, Cao J *et al*. Flexible Transparent Iontronic Film for Interfacial Capacitive Pressure Sensing. *Adv Mater* 2015; **27**: 6055-62.
3. Oldham KB. A Gouy–Chapman–Stern model of the double layer at a (metal)/(ionic liquid) interface. *J Electroanal Chem* 2008; **613**: 131-8.
4. Allagui A, Benaoum H, Olendski O. On the Gouy–Chapman–Stern model of the electrical double-layer structure with a generalized Boltzmann factor. *Physica A: Statistical Mechanics and its Applications* 2021; **582**: 126252.
5. Delamarche E, Schmid H, Michel B *et al*. Stability of molded polydimethylsiloxane microstructures. *Adv Mater* 1997; **9**: 741-6.
6. Qiu Z, Wan Y, Zhou W *et al*. Ionic Skin with Biomimetic Dielectric Layer Templated from Calathea Zebrine Leaf. *Adv Funct Mater* 2018; **28**: 1802343.
7. Lin I, Ou K, Liao Y *et al*. Viscoelastic Characterization and Modeling of Polymer Transducers for Biological Applications. *J Microelectromech S* 2009; **18**: 1087-99.
8. Cho SH, Lee SW, Yu S *et al*. Micropatterned Pyramidal Ionic Gels for Sensing Broad-Range Pressures with High Sensitivity. *Acs Appl Mater Inter* 2017; **9**: 10128-35.
9. Yoon SG, Park BJ, Chang ST. Highly Sensitive Piezocapacitive Sensor for Detecting Static and Dynamic Pressure Using Ion-Gel Thin Films and Conductive Elastomeric Composites. *Acs Appl Mater Inter* 2017; **9**: 36206-19.
10. Chhetry A, Kim J, Yoon H *et al*. Ultrasensitive Interfacial Capacitive Pressure Sensor Based on a Randomly Distributed Microstructured Iontronic Film for Wearable Applications. *Acs Appl Mater Inter* 2019; **11**: 3438-49.
11. Bai N, Wang L, Wang Q *et al*. Graded intrafillable architecture-based iontronic pressure sensor with ultra-broad-range high sensitivity. *Nat Commun* 2020; **11**.
12. Tay RY, Li H, Lin J *et al*. Lightweight, Superelastic Boron Nitride/Polydimethylsiloxane Foam as Air Dielectric Substitute for Multifunctional Capacitive Sensor Applications. *Adv Funct Mater* 2020; **30**: 1909604.
13. He X, Liu Z, Shen G *et al*. Microstructured capacitive sensor with broad detection range and long-term stability for human activity detection. *Npj flexible electronics* 2021; **5**: 1-9.
14. Shen Z, Zhu X, Majidi C *et al*. Cutaneous Ionogel Mechanoreceptors for Soft Machines, Physiological Sensing, and Amputee Prostheses. *Adv Mater* 2021; **33**: 2102069.
15. Xiao Y, Duan Y, Li N *et al*. Multilayer Double-Sided Microstructured Flexible Iontronic Pressure Sensor with a Record-wide Linear Working Range. *ACS Sens* 2021; **6**: 1785-95.
16. Kwon JH, Kim YM, Moon HC. Porous Ion Gel: A Versatile Ionotronic Sensory Platform for High-Performance, Wearable Ionoskins with Electrical and Optical Dual Output. *Acs Nano* 2021; **15**: 15132-41.
17. Ha KH, Zhang W, Jang H *et al*. Highly Sensitive Capacitive Pressure Sensors over a Wide Pressure Range Enabled by the Hybrid Responses of a Highly Porous Nanocomposite. *Adv Mater* 2021; **33**: 2103320.
18. Su Q, Zou Q, Li Y *et al*. A stretchable and strain-unperturbed pressure sensor for motion interference-free tactile monitoring on skins. *Sci Adv* 2021; **7**: i4563.
